# Distinct evolutionary patterns of tumour-immune escape and elimination determined by extracellular matrix architectures

**DOI:** 10.1098/rsif.2025.0116

**Published:** 2025-07-09

**Authors:** Yijia Fan, Jason T. George

**Affiliations:** 1 Department of Biomedical Engineering, Texas A&M University College Station, TX, USA; 2 Translational Medical Sciences, Texas A&M University Health Science Center, Houston, TX, USA; 3 Center for Theoretical Biological Physics, Rice University, Houston, TX, USA

**Keywords:** tumour evolution, extracellular matrix, epithelial-to-mesenchymal transition, tumour–T-cell interaction, Life Sciences–Mathematics interface, biomedical engineering, evolution, computational biology

## Abstract

Cancer progression remains a significant clinical challenge. Phenotypic adaptation by tumour cells results in disease heterogeneity, which drives treatment resistance and immune escape. T-cell immunotherapy, while effective at treating some cancer subtypes, can also fail due to limits on tumour immunogenicity or T-cell recognition. For example, one potential contributor to immune escape involves the density and alignment of the extracellular matrix (ECM) surrounding tumours, also known as tumour-associated collagen signature (TACS). However, the specific mechanisms by which aligned fibres contribute to decreased patient survival rates have not yet been decoupled. Here, we developed EVO-ACT (EVOlutionary agent-based cancer T-cell interaction), a two-dimensional agent-based modelling framework designed to investigate how different TACS architectures impact tumour evolution and dynamic interactions with CD8^+^ T cells. Our results highlight that TACS-driven modulation of T-cell dynamics, combined with phenotypic adaptation, such as epithelial-to-mesenchymal transition, underlies differences in tumour immunogenicity and the application of our model can successfully recapitulate clinically observed breast cancer survival trends.

## 1. Introduction

The immune system plays a central role in the adaptive response against tumour progression, wherein cytotoxic T cells (CD8^+^ T cells), hereafter referred to as T cells, attempt to engage with and eliminate the cancer population. This interaction results in immunoediting of tumour populations that can either lead to tumour escape, elimination or a sustained equilibrium period [1–5]. Prior experimental and theoretical work has been directed at understanding how repeated tumour–immune interactions affect the ultimate dynamics of cancer progression and escape [6–10]. These earlier models have described how clonally heterogeneous cancer populations evolve under adaptive immune selective pressures.

**Figure 1.**
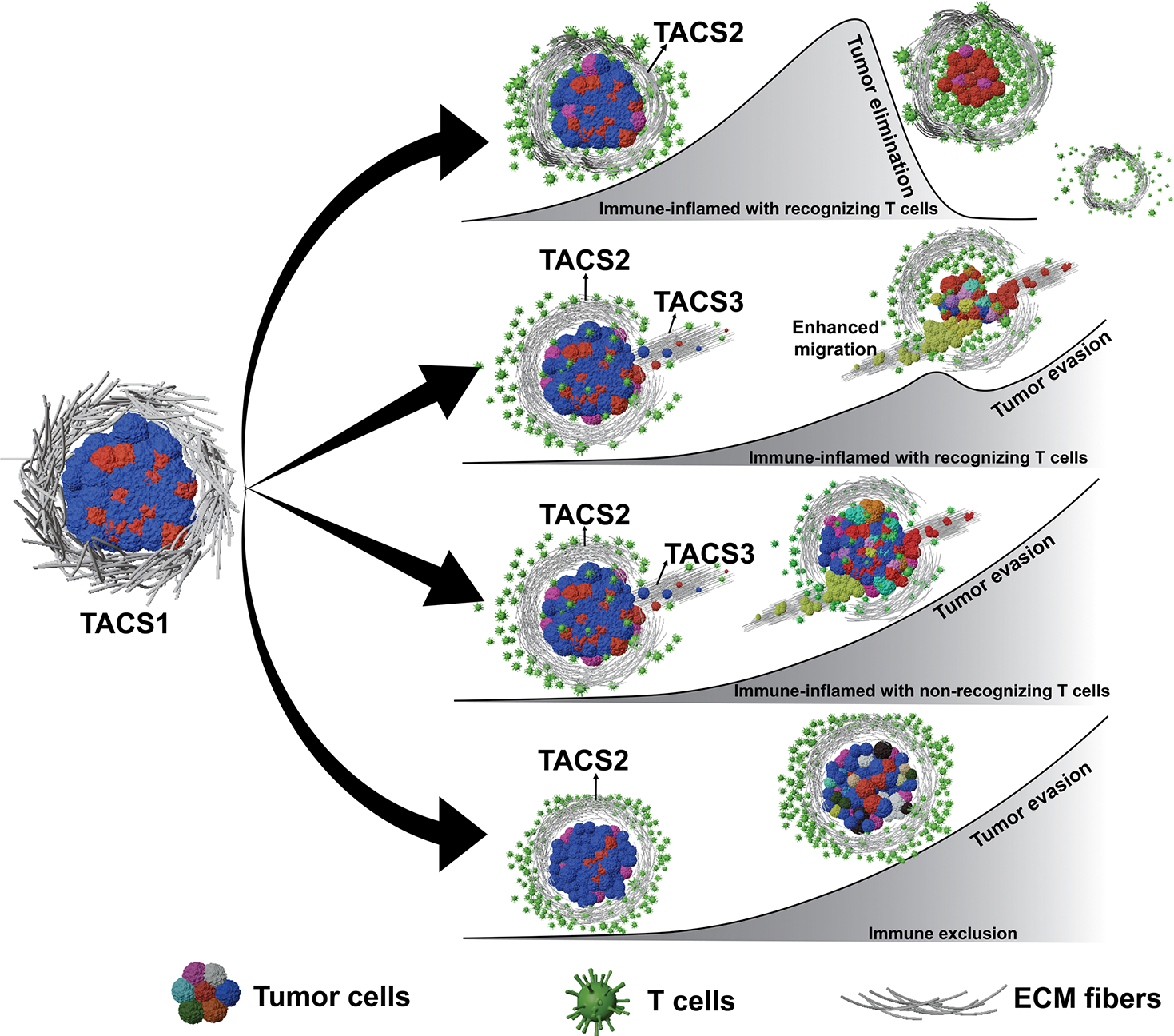
Tumour progression schematics under different TACS and T-cell infiltration scenarios. EVO-ACT considers tumours initially in TACS1, which then progress to TACS2 and TACS3. Under different immune infiltration conditions [15], interactions between T cells and cancer cells can give rise to tumour elimination (row 1) or escape (rows 2–4). Tumour heterogeneity arises through stochastic adaptation, resulting in variable TAA presentations. Different tumour-cell colours represent potential tumour heterogeneity.

It is now appreciated that the adaptive immune system can capably clear cancer in some cases, while in others, tumour-immune escape occurs. The immune microenvironment plays an important and multifaceted role in this process. One unanswered question relates to the role of ECM (extracellular matrix) organization and its effects on tumour-immune recognition. In solid malignancies, ECM geometry in the microenvironment has been associated with disease stage [11,12] and observed T-cell infiltration [13,14]. Specifically, empirically observed ECM topologies are frequently categorized based on fibre arrangement: random fibres (TACS1), circumferentially aligned fibres (TACS2) and radially arranged fibres (TACS3), illustrated in figure 1 [12]. Researchers have further refined this classification and identified up to eight distinct TACS subtypes, which build upon the foundational TACS1-3 framework by capturing more nuanced variations in fibre organization [16]. Additionally, different TACS can coexist within the same tumour and are not restricted to specific cancer stages or patients [17–19]. In breast cancer, TACS3 often emerges after TACS1 transitions to TACS2 [18]. Despite the fact that a clear negative correlation between TACS3 and breast cancer patient survival has been established [18], the specific roles and extent of TACS in sculpting T-cell-driven cancer evolution remain uncharacterized. Mechanisms underlying how TACS influences cell movement are still not fully elucidated, with divergent opinions on the precise details involving how and to what extent the ECM mediates immune cell infiltration [13,14,20–22]. At present, we lack a physical model relating the impact of TACS on the spatial coevolution between an adaptive immune repertoire and a heterogeneous population of evading cancer cells.

To address these complexities, we developed EVO-ACT (EVOlutionary agent-based cancer T-cell interaction), a spatially explicit agent-based modelling framework that captures the dynamic interaction between tumour and T cells in different TACS signatures. Unlike existing models that focus on isolated processes, EVO-ACT integrates tumour–immune–ECM dynamics into a unified system, enabling spatiotemporal analysis of how TACS structures and phenotypic adaptation shape T-cell infiltration, immunoediting and immune escape. This represents, to our knowledge, the first stochastic agent-based model of spatially dependent tumour–immune interactions based on T-cell recognition of tumour antigens. Our modelling framework is capable of generating dynamical insights into how local ECM remodelling and antigen-driven T-cell behaviour jointly influence immune surveillance and tumour evolution.

Our results suggest that the degree of cancer immunoediting is dependent on TACS-specific differences in T-cell infiltration and moving efficiency, and that TACS have a greater impact on chemokine-directed T-cell infiltration than they do on tumour-immune evasion. When applied to predict differences in TACS3-dependent disease progression, we find that our modelling framework requires the inclusion of additional phenotypic adaptation mechanisms, such as the epithelial-to-mesenchymal transition (EMT), in order to successfully recapitulate clinically observed cancer survival trends. Our model predicts that immunogenicity differences through decreased tumour-associated antigen (TAA) availability and immune checkpoint upregulation synergize to result in immune escape, which successfully predicts overall survival trends in breast cancer [23,24]. The EVO-ACT framework provides a detailed dynamical description of the role of TACS in tumour evolution when subject to adaptive immune selective pressure. We anticipate that its use can be more broadly applied to understand cancer evolutionary patterns and treatment success or failure in specific cases where observed TACS architecture and phenotypic status are previously defined.

## 2. Model development

To investigate the dynamic interplay between tumour cells, T cells and the ECM, we developed EVO-ACT, a spatially explicit agent-based model. EVO-ACT simulates tumour growth, immune infiltration and ECM remodelling over time, allowing for mechanistic exploration of how structural and phenotypic heterogeneity influences cancer–immune interactions and tumour elimination or escape. Below, we briefly outline the modelling approaches used to represent tumour-cell behaviour, T-cell dynamics and ECM structure. We refer readers to §5 for full details.

### 2.1. Cancer cells

To model cancer cells, we assumed that a small collection (*n* ~ 4000) corresponding to a total tumour diameter of 0.1 cm of tumour precursors reside at the centre of a circular region of interest, from which active tumour cells may extravasate outward, invade and divide [25]. These tumour cells, together with T cells, comprise agents in our model. We assumed that cancer cells may divide and migrate at per-cell rates of *λ* and *α*_t_, respectively. Cancer cells are also assumed to possess an initial collection of shared TAAs displayed on their surface. For foundational understanding, we generated TAAs randomly according to a Poisson (v=100) distribution. Each cell may also undergo phenotypic adaptation with a rate of $\mu =5\times 10^{-4}$ per cell division. We assumed that each adaptation event results in an equal likelihood of the addition or removal of a Poisson number of TAAs with a mean of 10, and that each TAA addition or removal increases or decreases the division rate by 1% of λ with equal probability. Evolution in this model results in distinct cancer populations, or ‘clones’, which are distinguished only based on their expression of TAAs and may occur through genetic or epigenetic adaptation mechanisms. We accounted for the ‘contact guidance’ theory of migration by assuming that each cancer cell randomly selects a fibre within five cell diameters (75 μm) along which to migrate [12], and equal probabilities are given to both fibre directions. We also considered variability in the starting division rates and TAA abundances.

In simulations that incorporate EMT, we assume that EMT occurs once a certain total tumour burden is achieved. Upon reaching this threshold, all dividing cells at the tumour periphery are assumed to undergo EMT, which was modelled by a decreased division rate (fivefold reduction), increased migration rate (fivefold increase), and decreased immunogenicity (reduction of TAAs by a Poisson-distributed random variable with mean of 15) to match previous experimental observations [24,26].

### 2.2. T cells

T cells comprise the other active agents in our modelling framework, and we modelled their dynamics by starting with an initial population of ($N_{T}=5000$) T cells located at the boundary of the region of interest [27]. These T cells migrate inward at a deterministic rate of $\alpha_{T}$. Their directed migration is influenced by the surrounding extrinsic microenvironment, which includes collagen fibre alignment and chemotaxis [12,28]. To model differential antigen specificities of distinct T-cell clones, we assumed that distinct T-cell receptors (TCR) exist (*Q* ≤ 5000), each having the capability to recognize various antigen signatures. In this model, T-cell diversity is characterized by the absolute number of distinct antigen specificities the T-cell population can cover, which correlates directly with the number of TAA-specific clones (q). T-cell recognition is thus a function of both Q and q. To model recognition, each migrating T cell surveys the vicinity (45 μm). Upon encountering at least one recognizable TAA, T cells eliminate the tumour cell and subsequently divide. To account for lineage-specific T-cell contractions in the absence of antigen signalling, daughter T cells possess a finite survival window, after which they are removed if they cannot recognize and eliminate another cancer cell (see electronic supplementary material, figure S1, for full details). While we do not explicitly include memory T cells in our model, our assumptions on T-cell dynamics maintain a small population of effector T-cell clones that have previously recognized TAAs.

We utilized a Gillespie simulation-based approach to model cancer and T-cell dynamics.

### 2.3. Extracellular matrix topology

We modelled ECM fibres situated outside the tumour core with normally distributed lengths (meanμ=10μm; variance $\sigma^{2}=\mu /10$) [29]. To reflect common patterns observed in some solid tumours, including breast tumour [18,30,31], fibre density was assumed to decrease radially outward from the tumour centre and initially all fibres are randomly oriented, corresponding to TACS1 [32]. To account for tumour-driven TACS remodelling [12], we allowed for each dividing tumour cell at the periphery to alter the fibre direction from TACS1 to TACS2 within a neighbourhood of the cell (75 μm). Based on previous findings, tumour cells can remodel fibres into a radial pattern within a fivefold neighbourhood relative to tumour radius [33]. For simplicity, based on the size of the central tumour, we assumed that dividing cells at the tumour boundary will collectively remodel fibres within approximately 0.18 cm into TACS3. We also studied the role of heterogeneous fibre alignment by adjusting the variance of fibre orientation in each of TACS2 and TACS3.

We refer the reader to electronic supplementary material, figure S2, for the initial spatial distribution of tumour, T cells and fibres in our model and §5 for full model details.

## 3. Results

### 3.1. Cancer evolution generates tumour-associated collagen signature-specific spatial signatures in the absence of immune selective pressure

**Figure 2.**
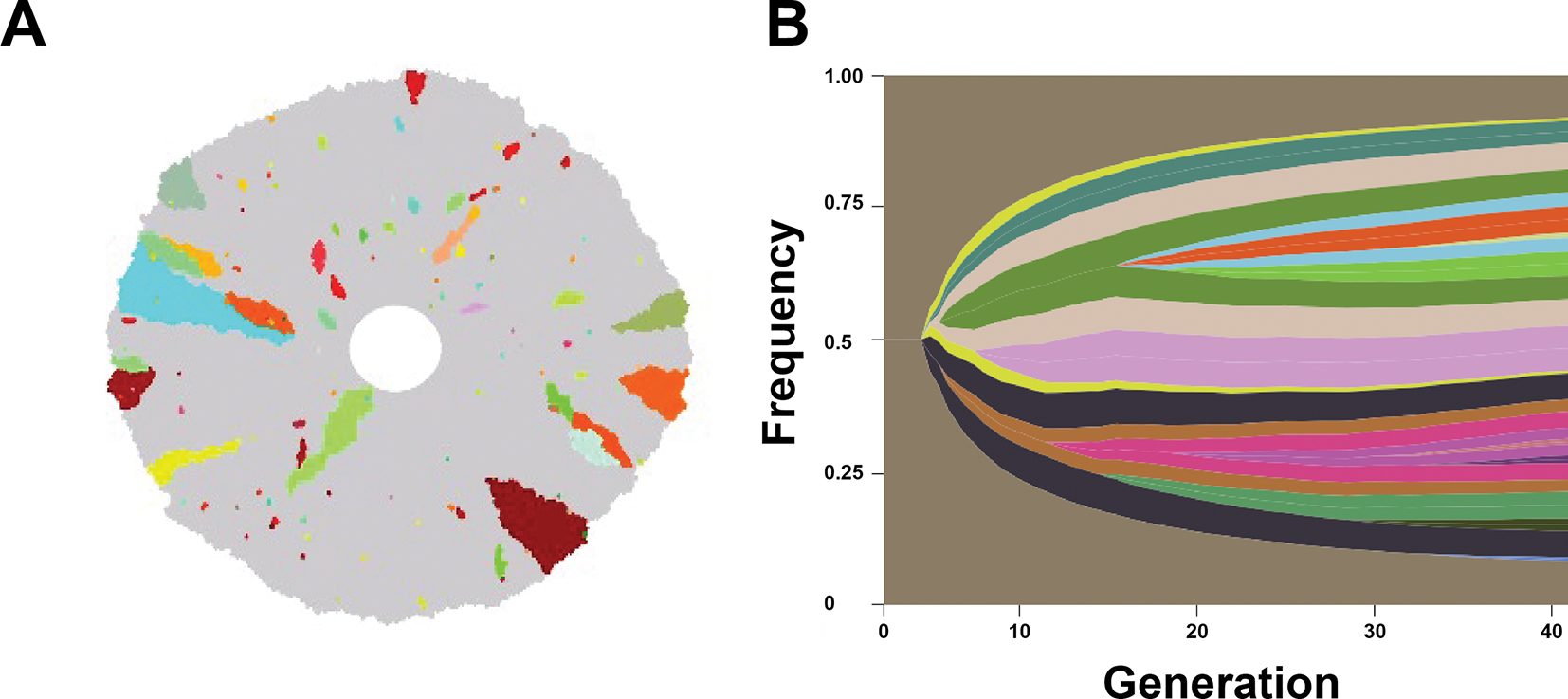
Spatial heterogeneity obtained through cancer evolutionary progression. (A) The spatial distribution of cancer clones is illustrated following progression to approximately 0.5 cm diameter (corresponding to approx. 110 000 tumour cells). Grey cells represent daughter cells arising from the founder clone, and additional colours distinguish subsequent clones that harbour distinct antigen expression patterns. (B) A representative fish plot indicating the frequency of each antigenic clone, where the brown represents the initial clone. The vertical axis represents the frequency of each tumour clone and the horizontal axis represents the generation.

In our model, tumour populations grow and acquire alterations that enhance their division rate, and also affect their antigenic burden. In the absence of T-cell recognition, cancer cells exhibited evolutionary trajectories comprised of outward growth and clones with increased fitness through enhancements in their growth rates are positively selected. Figure 2A represents one stochastic realization of this process for a single tumour population that divides until reaching a diameter of approximately 0.5 cm. Our results capture the spatial variability in tumour heterogeneity and are in qualitative agreement with prior models in the regime of tumour growth and invasion into neighbouring tissue (figure 2B) [34].

**Figure 3.**
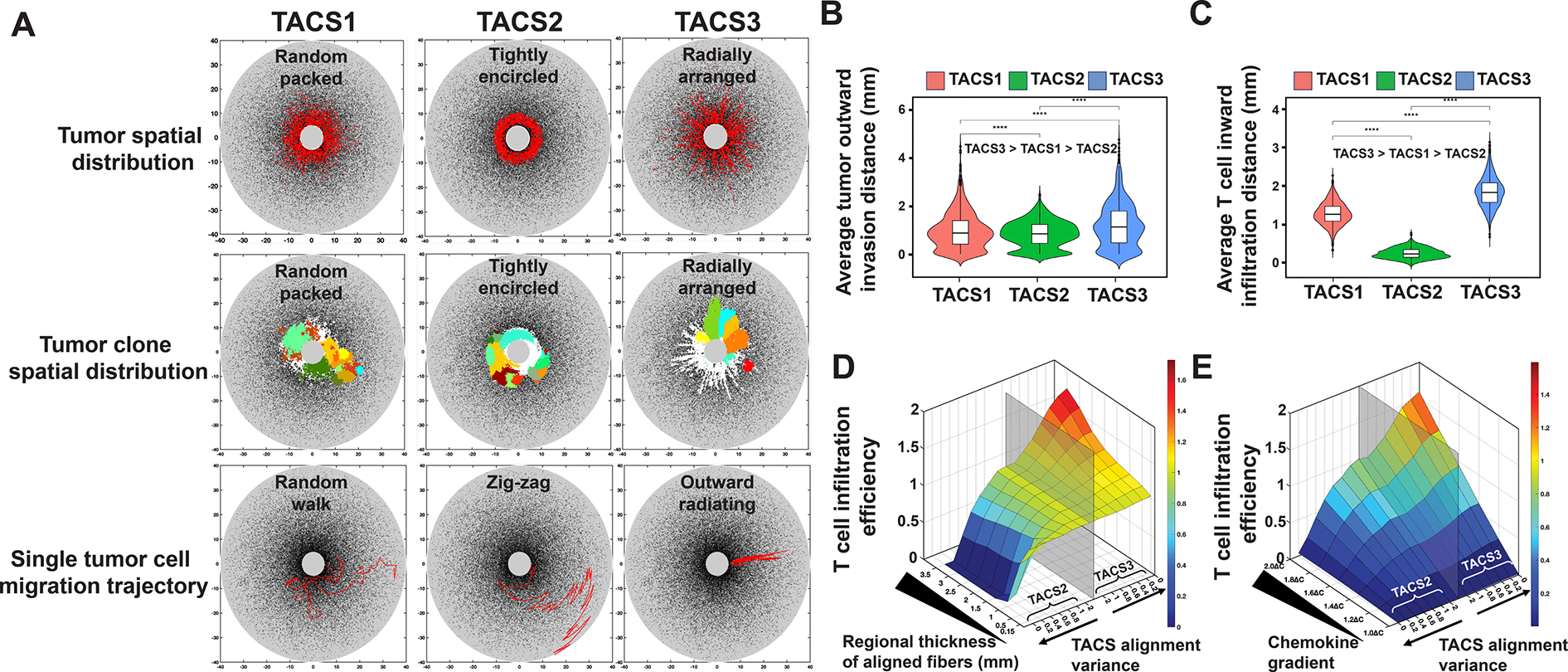
TACS influences spatial distribution, migration direction and efficiency of both tumour cells and T cells, with chemical attractant amplifying TACS impact on T-cell infiltration efficiency. (A) Tumour spatial distribution (λ=0.1, $\alpha_{t}=0.01$, $\alpha_{T}=0$), antigenic clonal variant spatial distribution (λ=0.1, $\alpha_{t}=0.01$, $\alpha_{T}=0$) and single-cell migration trajectories (λ=0, $\alpha_{t}=0.1$, $\alpha_{T}=0$) in TACS1–3. In the single tumour-cell migration trajectory, the Δt values in TACS1–3 are 0.33, 0.75 and 0.15, respectively. (B) Average tumour invasion distance ($N_{t}=10^{3}$ tumour cells) across TACS1 to TACS3 within a fixed time frame. TACS2 and TACS3 are perfectly aligned (alignment variance σ=0 for each orientation). ****p < 0.0001. $\lambda =0,\;\alpha_{t}=0.1,\;\alpha_{T}=0$. (C) Average T-cell infiltration distance ($N_{T}=10^{3}$ T cells) across TACS1 to TACS3 within the consistent time frame depicted in (B) TACS2 and TACS3 are perfectly aligned (alignment variance σ=0 for each orientation)). ****p < 0.0001. $\lambda =0,\;\alpha_{t}=0$, $\alpha_{T}=0.1$ (D) Normalized T-cell infiltration efficiency in TACS1–3 with varying alignment region thickness and alignment variance. We measured the time taken for $N_{T}=500$ T cells to infiltrate from the boundary of the region of interest to the boundary of the central tumour circle under varying thicknesses of the alignment region and varying alignment variances of TACS2 and TACS3. This time was normalized against the time spent in TACS1. The semi-transparent grey plane in the graph represents the baseline or T-cell infiltration efficiency in TACS1. The left side of this plane represents the infiltration efficiency of T cells in TACS2, with TACS2 features becoming more pronounced as one moves further to the left. Conversely, the right side of this plane represents the infiltration efficiency of T cells in TACS3, with TACS3 features becoming more pronounced as one moves further to the right. The *y*-axis represents the thickness of the alignment region, with thicker regions positioned toward the inner side. $\lambda =0,\;\alpha_{t}=0,\;\alpha_{T}=0.1$. (E) Normalized T-cell infiltration efficiency in TACS1–3 with varying chemokine gradients and alignment variances σ. We replicated the experiments in (D), with a default aligned region thickness of 0.5 mm, and replaced the regional thickness of aligned fibre with chemokine gradients. $\lambda =0,\;\alpha_{t}=0,\;\alpha_{T}=0.1$.

Given the well-established clinical relevance of TACS [11,12,16,18,35,36], we next aimed to identify how TACS impacts cancer patient survival or tumour elimination and escape. To investigate this, we first explored how TACS influences the encounter between tumours and T cells. Based on the ‘contact guidance’ theory [37], we hypothesized that TACS affects the spatial distribution and migration of cells. To test this, we simulated an initially small collection of tumour cells dividing within the three types of TACS in the absence of immune pressure, with TACS2 and TACS3 being perfectly aligned, alignment variance σ=0 for each orientation. For each TACS architecture, a distinct pattern of tumour spatial distribution emerged: tumour cells are randomly packed in TACS1, they are tightly encircled in TACS2 and radially arranged in TACS3 (figure 3A). These patterns also manifest in corresponding random (TACS1), circumfrential (TACS2) and radial (TACS3) spatial distributions of tumour clones (figure 3A). Subsequent analysis of single-cell cancer migration trajectories also revealed differences in their motion, with a contact-guided random-walk pattern in TACS1, an outward ‘zig-zag’ motion in TACS2 and an ‘outward radiating’ motion in TACS3. Together, these results highlight how distinct TACS can significantly influence cancer cell migration and the subsequent spatial heterogeneity observed across otherwise identical underlying tumour evolutionary processes. We postulated that such differences may be relevant for immune recognition since, for example, the surface area of the tumour boundary in TACS3 is substantially larger than in TACS2 with fewer subclones protected in the tumour interior (electronic supplementary material, figure S3).

### 3.2. Tumour-associated collagen signature generates distinct patterns of cell migration and consequent cancer immunoediting

#### 3.2.1. Tumour-associated collagen signature determines cancer and T-cell migration efficiencies

To quantify the role of TACS in cancer and T-cell migration, we next tracked migration by simulating ($N_{t}=10^{3}$) tumour cells undergoing contact-guided migration along ECM fibres and $\left(N_{T}=10^{3}\right)$ T cells undergoing directed migration towards a chemokine signal over a fixed time period. In each case, we maintained perfect alignment of TACS2 and TACS3 (alignment variance σ=0) for comparison purposes. Our simulations predict that migration efficiencies in our model are maximal in TACS3, intermediate in TACS1 and minimal in TACS2 for both T cells and tumour cells (figure 3B,C). These findings are consistent with the fact that radially oriented TACS3 fibres direct T-cell movement to the primary tumour mass, while TACS2 results in circumferential movement that reduces the overall migration rate observed in randomly oriented TACS1 fibres. Despite similar trends in the relative cancer and immune cell movements for each TACS, these differences were substantially more pronounced for T cells than for cancer cells (figure 3B,C). These results suggest that the benefit to migration of TACS3 significantly favours T-cell infiltration over cancer escape.

To better understand the role of TACS fibres and cell signalling on T-cell migration, we performed additional simulations tracking the migration efficiency of ($N_{T}=500$) T cells from the region of interest boundary to the tumour centre boundary under TACS1–3. We also independently varied the regional thickness of aligned fibres and the strength of the chemokine gradient guiding T-cell migration. Migration efficiency was taken to be the inverse of mean migration time, and each result is normalized to the mean values obtained for TACS1. Our results (figure 3D,E) illustrate the spectrum on which highly versus loosely aligned TACS influence T-cell migration. Larger regions of TACS fibres amplify the observed TACS-specific migration differences, and a larger chemokine gradient consistently enhances infiltration efficiency across all TACS conditions. Moreover, our results show that TACS2 does not constitute an absolute barrier to T-cell infiltration; rather, it diminishes the efficiency of T-cell infiltration. The degree to which this efficiency is diminished relies on factors such as the degree of alignment and thickness of the TACS2 region. Our findings support previous work showing that clinically observed patterns of T-cell exclusion are consistent with differences in T-cell chemical signalling rather than a physical barrier to T-cell infiltration [21].

We also conducted a sensitivity analysis of tumour migration and T-cell infiltration efficiency (electronic supplementary material, figure S4). The results indicate that the migration rate of tumour cells has the greatest impact on migration efficiency. The alignment of the ECM, or various TACS, has a relatively minor effect on tumour migration efficiency. Variations in ECM density across different TACS areas show differential impacts. The density of the ECM has a negligible impact on tumours in TACS1 and TACS3; however, for tumours in TACS2, the combined effect of TACS2 and high-ECM density significantly impedes tumour-cell migration. Regarding T-cell infiltration efficiency, the migration rate shows a substantial effect and the impact of chemokine gradients in TACS3 is notably more influential compared with other TACS. Different TACS have a more pronounced impact on T-cell infiltration than on tumour-cell migration. Similarly, the ECM density also affects T-cell infiltration, with varying effects in different TACS areas. Our findings suggest that reducing the ECM density in the TACS2 region shows the most significant enhancement in T-cell infiltration efficiency.

#### 3.2.2. Tumour-associated collagen signature-specific tumour spatial heterogeneity and T-cell infiltration together generate variety in cancer immunoediting

**Figure 4.**
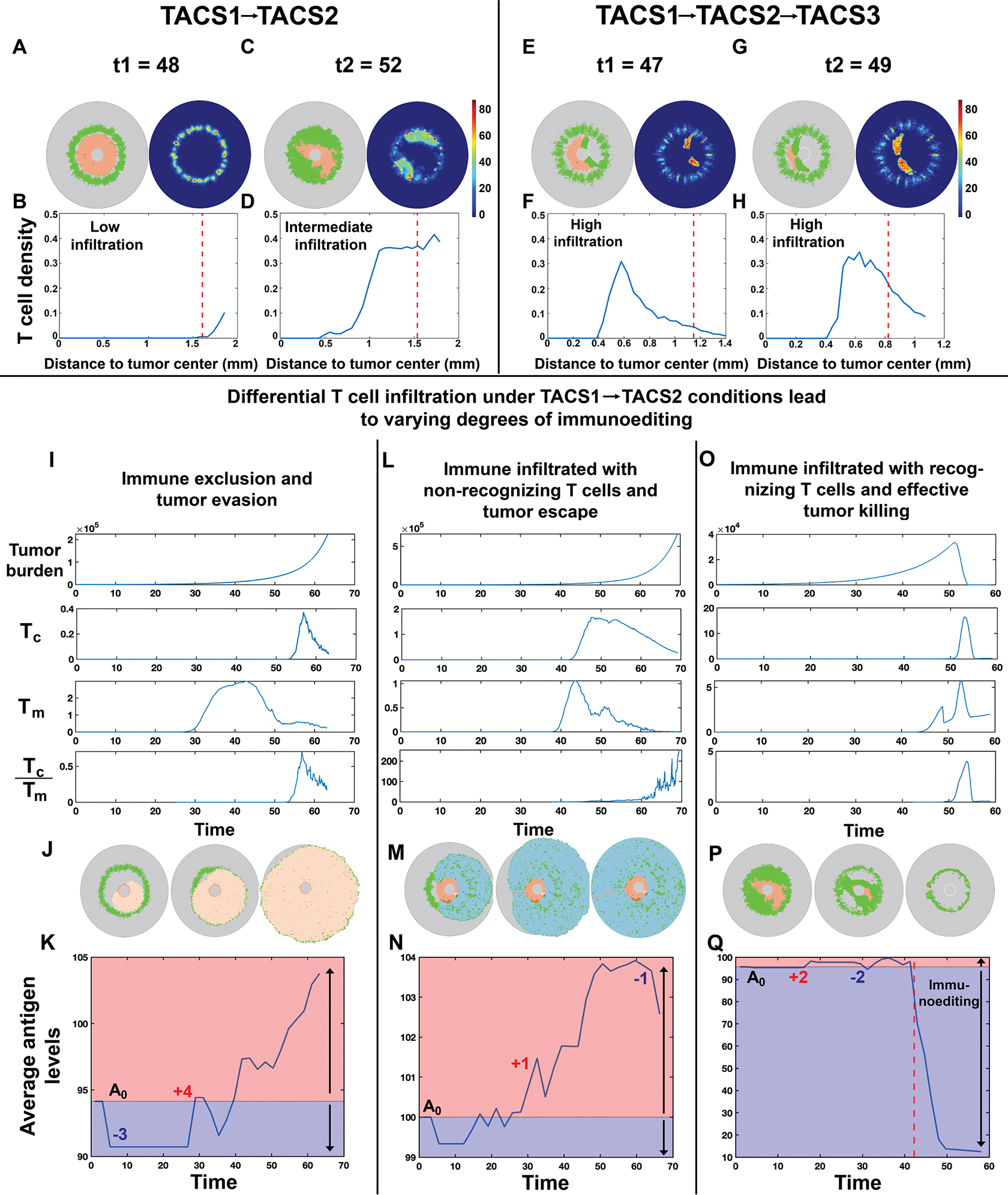
TACS impacts spatial heterogeneity and infiltration levels of T cells, consequently influencing immunoediting. TACS3 promotes more efficient T-cell infiltration. (A,C,E,G) Illustrative examples of tumour–T-cell interaction snapshots and associated T-cell density heatmaps within TACS1→TACS2 (A,C) and TACS1→TACS2→TACS3 (E,G) conditions at t1 and t2 time points. (B,D,F,H) The radial distribution of T-cell density from the tumour centre to the boundary of the region of interest is depicted. The red dashed line represents the boundary of the tumour core in the above two conditions, Q=210,q=10. (I,L,O) Tumour burden and T-cell density were quantified in the tumour core ($T_{c}$) and margin ($T_{m}$), and $T_{c}:T_{m}$. In immune exclusion and non-recognizing T-cell inflammation conditions, Q=210,q=0. In recognizing T-cell inflammation condition, Q=210,q=10. (J,M,P) Snapshots of tumour and T-cell interactions in three T-cell infiltration conditions. T cells are represented as green dots. The remaining colours denote tumour cells. (K,N,Q) The average antigen levels were analysed for each of the three conditions across 10 iterations. In each plot, initial antigen levels ($A_{0}$) partition the graph into red (antigen gain) and blue (antigen loss) segments. Higher *y*-values indicate higher antigenicity. The red dashed line in (Q) marks the onset of widespread tumour killing or immunoediting by T cells. In all conditions, $\lambda =0.1,\;\alpha_{t}=0.02,\;\alpha_{T}=1.8$.

We next sought to understand how TACS-dependent spatial distributions in cancer clones and variable T-cell infiltration efficiencies together affect tumour elimination and escape. Given that T-cell infiltration is often correlated with greater patient overall survival across various tumour subtypes [38–40], we first quantified the extent of T-cell infiltration in TACS environments by comparing the spatial distribution of T-cell density in the tumour core and tumour margin to capture different immune infiltration patterns and assess how ECM architecture may influence immune accessibility. Since our model does not include other cell types present in the tumour microenvironment, the tumour core is defined as the area enclosed by the current tumour boundary, while the tumour margin is immediately exterior to the boundary of the tumour core and extends outward by 250 μm [21]. Given that both TACS3-positive and TACS3-negative environments are commonly observed in clinical breast cancer [18], we performed stochastic simulations of tumour growth, T-cell infiltration and eventual tumour–T-cell interactions for TACS1→TACS2 (figure 4A–D) and TACS1→TACS2→TACS3 (figure 4E–H) conditions. We refer readers to §5 for details on TACS notations. In each case, we calculated an average T-cell density in the tumour core relative to the tumour margin to differentiate infiltrating and non-infiltrating T cells at the tumour boundary (indicated by the red dashed lines in figure 4B,D,F,H).

We observed distinct profiles of T-cell infiltration in TACS1→TACS2 (figure 4A–D) and TACS1→TACS2→TACS3 (figure 4E–H) conditions. Heat maps depicting T-cell densities indicate regions where active T-cell recognition and concomitant expansion occurs (figure 4A,C,E,G). In particular, TACS1→TACS2→TACS3 environments are characterized by high T-cell infiltration into the tumour core relative to the tumour margin. These findings contrast with TACS1→TACS2 environments, which were characterized by low-to-intermediate levels of T-cell infiltration (figure 4B,D,F,H). These trends were maintained in additional simulations that assumed perfectly aligned TACS2 and TACS3 signatures (alignment variance σ=0) (electronic supplementary material, figure S5). The observation that enhanced infiltration and consequent T-cell recognition occurs in a TACS3 environment is consistent with the prior finding that TACS3 provides greater benefit to T-cell mobility [41,42]. Moreover, reductions in T-cell infiltration in TACS2 still permit T-cell recognition and do not function as an absolute barrier to recognition, even in perfectly circumscribed tumours (electronic supplementary material, figure S5). Finally, greater TACS2 thickness enhances the inhibitory capacity on T cells, thereby promoting tumour evasion (electronic supplementary material, figure S6).

Given these differences in T-cell infiltration, we next sought to observe how TACS-specific effects on cell migration and tumour heterogeneity together impact immunogenicity during tumour progression. We performed additional simulations to assess TACS1→TACS2 T-cell infiltration in greater detail. We first modelled an immune-excluded phenotype by confining 95% of T cells to the tumour margin. These simulations were compared against cases that allowed for T-cell infiltration. In all conditions, we tracked T-cell infiltration into the tumour core ($T_{c}$), tumour margin ($T_{m}$) and the ratio $T_{c}:T_{m}$ (figure 4I,L,O). We subsequently repeated 10 replicates for each condition, and average tumour antigenic burden was calculated over time in each case (figure 4K,M,Q). TACS1→TACS2 cases resulted in both immune-excluded and immune-infiltrated tumours (figure 4I–Q), and only a subset of cases with T-cell infiltration exhibited significant T-cell expansions, which we distinguish as ‘non-recognizing’ (figure 4L–N) or ‘recognizing’ (figure 4O–Q). In both immune-excluded and infiltrated non-recognizing cases, we observed a steadily increasing average antigenic burden during cancer progression, which is comparable to empirically observed mutation accumulation rates (figure 4K,N) [43,44]. This behaviour contrasted with the infiltrated recognizing case, wherein TAA availability notably declined over time and is consistent with strong immunoediting (figure 4O–Q). Our model predicts that active immune pressure results in tumour clones that tend to become less immunogenic over time, suggestive of the strong selective pressure operant during T-cell infiltration and in agreement with previous findings [2,45]. Under T-cell-mediated predation, the temporal dynamics of tumour progression and clonal evolution are presented in electronic supplementary material, figure S7. We further simulated the progression of two tumour types with differing immunogenicity under the same TACS context, corresponding to clinically observed progressively ‘hot’ and progressively ‘cold’ tumours (electronic supplementary material, figure S8) [46]. These dynamics underscore that, in our model, T-cell infiltration alone is insufficient to predict T-cell response, with spatially dependent T-cell expansion and tumour immunogenicity being more indicative of effective recognition and tumour elimination (electronic supplementary material, figure S9).

### 3.3. Tumor-associated collagen signature 3-associated phenotypic adaptation decreases predicted survival rates and impairs the efficiency of checkpoint inhibitor therapy

#### 3.3.1. Tumor-associated collagen signature 3-associated phenotypic changes are predicted to drive reductions in survival rates

**Figure 5.**
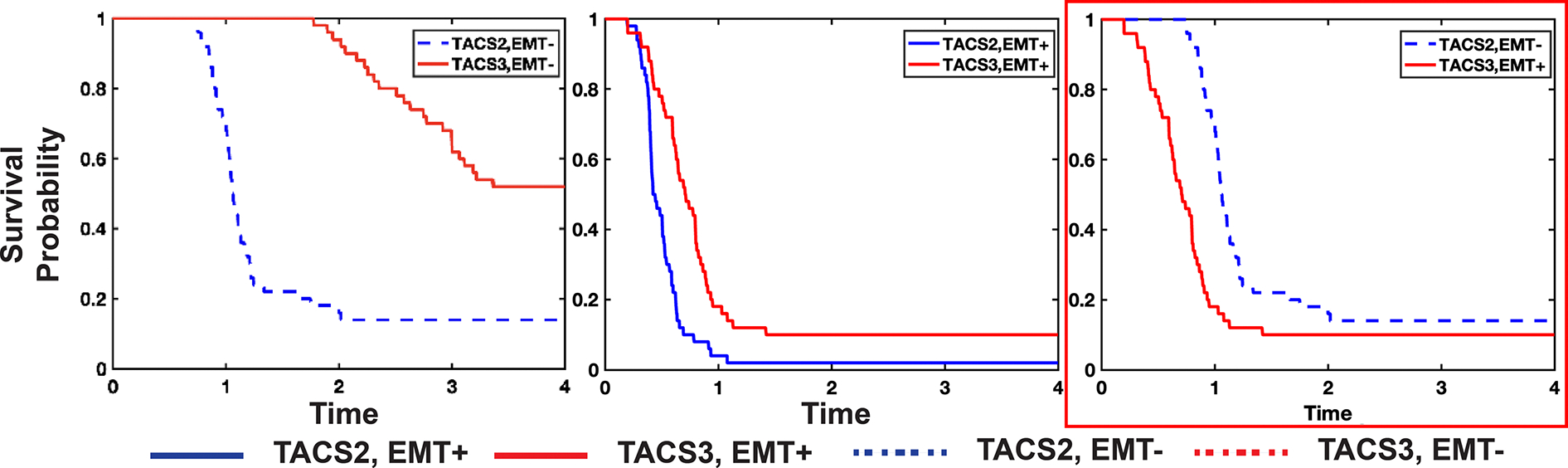
The concurrence of TACS3 and tumour adaptive changes is a significant factor contributing to the lower survival rates in TACS3 cases. The survival probability from 50 repeated experiments with varying TCR diversity in TACS2/EMT− (median survival time = 1.04), TACS3/EMT− (median survival time = 2.578) (A), TACS2/EMT+ (median survival time = 0.423), TACS3/EMT+ (median survival time = 0.656) (B), TACS2/EMT−, TACS3/EMT+ (C). In all simulations, we assumed that clinical immune escape occurs when the tumour size reaches approximately 0.5 cm. *p* ≤0.001. The red box marks the simulation condition (EMT after TACS2–TACS3 transition) that best matches observed clinical survival trends. To simplify, we reduced the region of interest size from a radius of 0.4 cm to 0.25 cm, meaning that the initial distance of T cells from the tumour is closer. $\lambda =0.4,\;\alpha_{t}=0.01,\;\alpha_{T}=1.6,\;\mu =8\times 10^{-4},\;Q=500-2500,\;q=100-500$.

Our previous results suggested that TACS3 in isolation confers an overall net benefit to immune recognition (figure 3C), an unexpected result in light of the fact that survival rates in TACS1→TACS2→TACS3 conditions are generally lower than those observed for TACS1→TACS2 conditions [18]. To investigate this further, we performed 50 stochastic simulations of tumour progression and immune recognition in perfectly aligned TACS2 and TACS3 conditions, respectively, each characterized by varying TCR specificity. In our model, a tumour size of approximately 0.5 cm (approx. 110 000 cells) was used as a threshold for local progression beyond immune control, with recorded progression times. We initially kept all other parameters constant while only altering TCR specificity (refer to §5 for full details). Despite exploring various parameter regimes (refer to §5 for full details), we consistently found that the survival rate of TACS3 was higher than that of TACS2 (figure 5A). Given that TACS3 typically occurs in the late stages of breast cancer [18], we reasoned that TACS3 cases may be accompanied by additional cancer cell phenotypic changes that are known to occur as the disease progresses [47]. One of the most well-established phenotypic changes in many epithelial cancers, including breast cancer, is the occurrence of the EMT [24,26,48]. EMT is modelled by enhanced tumour migratory capacity, reduced proliferative ability and decreased immunogenicity. Introducing EMT separately in TACS2 and TACS3, we observed higher overall survival rates in TACS3 compared with TACS2 (figure 5B; electronic supplementary material, figure S10A). Our simulations that include EMT only after TACS2–TACS3 progression most closely match clinically observed outcomes (figure 5C) [18], consistent with the fact that TACS3 and EMT are both likely to occur in later disease. Modelling TACS and EMT in this coupled way is also consistent with experimental evidence demonstrating that ECM influences EMT regulation, and reciprocally, EMT induces changes in ECM remodelling [47,49–52]. Moreover, by considering population-level variability in TCR specificity (see §5 for full details) for large-scale simulations of survival, we were able to identify parameters which more accurately reflect observed survival trends in TACS (electronic supplementary material, figure S11).

**Figure 6.**
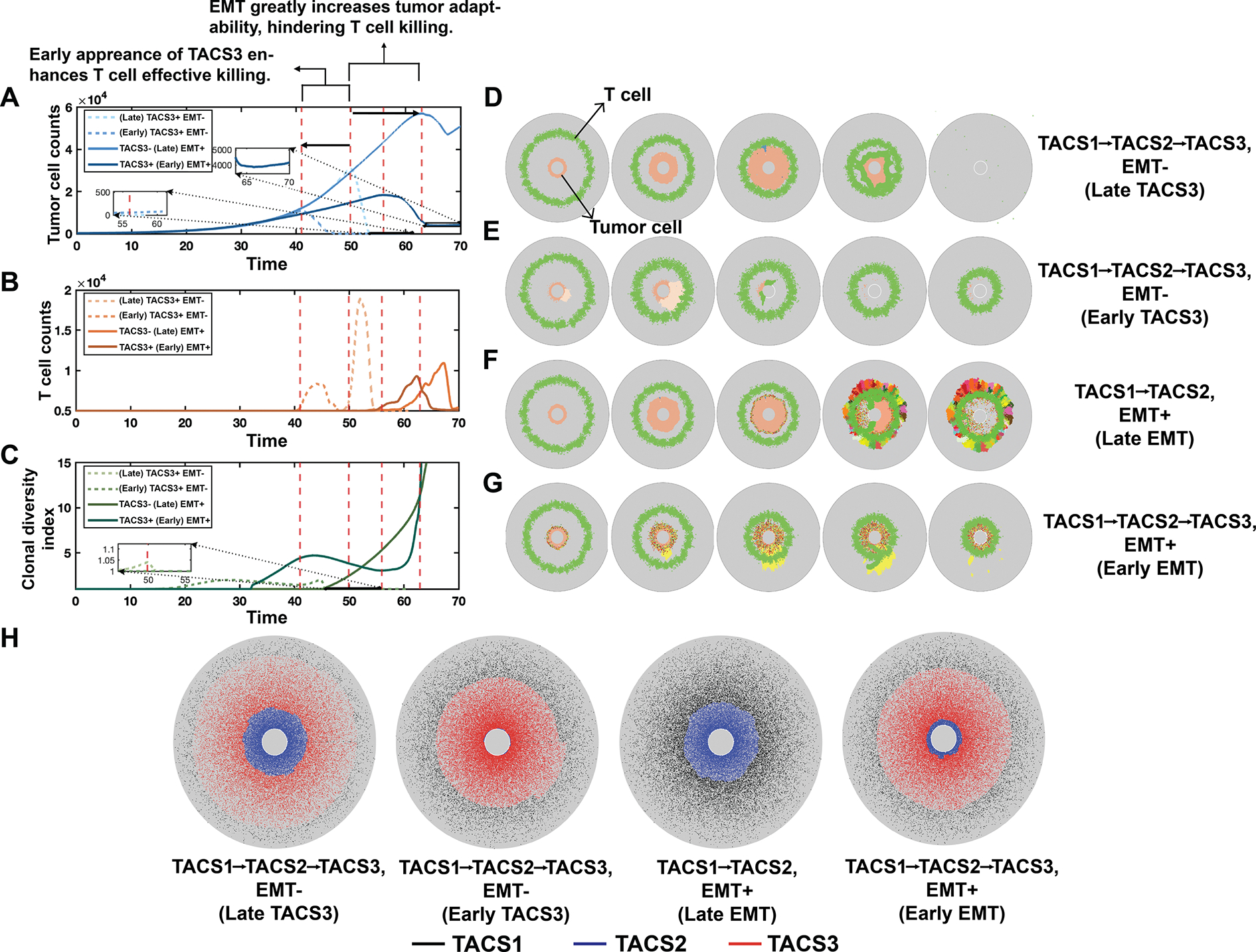
Tumour and T-cell interaction dynamics in the presence or absence of TACS3 and EMT. (A) Tumour burden is depicted over time. The red dashed lines represent the time at which T cells initiate large-scale tumour killing in each condition. (B) T-cell counts are depicted over time. (C) Clonal diversity index is depicted over time. See §5 for further details on the clonal diversity index. To simplify visualization, conditions with TACS1 → TACS2 ECM alignment were labelled as ‘TACS3−’ while those including all three patterns (TACS1 → TACS2 → TACS3) were labelled as ‘TACS3+’ in (A–C). (F–G) Snapshots of each condition at different times, respectively. T cells are represented by green, while the remaining cells represent tumour cells, with different colours indicating distinct tumour clones. Among them, (D) TACS3 occurs without EMT, and TACS3 occurs early. (E) TACS3 occurs without EMT, and TACS3 occurs late. (F) EMT occurs without TACS3. (G) TACS3 and EMT occur simultaneously. (H) Final distribution of fibres in each condition. TACS1 fibres are marked with black, TACS2 fibres are marked with blue, and TACS3 fibres are marked with red. In all conditions, λ=0.1, $\alpha_{t}=0.02$, $\alpha_{T}=1.8$, Q=210, q=10.

To better understand the respective impacts of TACS3 and EMT on disease progression, we varied the occurrence of EMT and the timing of TACS3 initiation. We first modified the timing of TACS3 occurrence, which from our earlier findings was expected to prolong T-cell residence times and infiltration efficiency (figure 6D,E). We observed significant reductions in tumour burden with early TACS3, along with an earlier occurrence of substantial tumour killing (figure 6A,B). While late TACS3 exhibits a higher peak in tumour burden, resulting in greater T-cell expansion compared with early TACS3 (figure 6B), the earlier onset of tumour killing in early TACS3 limits the time available for tumour evolution. These observations suggest that TACS3: (i) facilitates enhanced T-cell infiltration, which is consistent with our previous findings, (ii) curtails the window for tumour evolution, and (iii) partially alleviates challenges associated with tumour eradication. Hence, by controlling the timing of TACS3 emergence, we did not observe significant challenges to the immune system. Even when TACS3 appeared later, the efficient infiltration facilitated by TACS3 could assist T cells in encountering cognate antigens (figure 6D).

Given that TACS3 alone cannot fully explain the clinically observed survival in breast cancer (figure 5A), we next assessed the impact of EMT occurring alongside TACS3. We identified three differences in EMT− (figure 6D,E) versus EMT+ (figure 6F,G) cases. First, tumour burden is notably higher in EMT+ cases. Second, the time at which tumour burden begins to significantly decrease is later in EMT+ cases. Third, there is a less dramatic decrease in tumour burden in EMT+ cases. These observations can be explained by higher levels of TAA-specific heterogeneity, reduced immunogenicity and enhanced migration in EMT+ cases, all of which impair T-cell killing. TACS3 enhances T-cell infiltration relative to TACS2 cases (figure 6A–F), thereby leading to earlier encounters between T cells and tumours and reductions in tumour burden (figure 6F–G). Collectively, these results suggest that phenotypic adaptation occurring late in TACS3 poses significant challenges to the immune system and reduces cancer elimination rates. From this, we conclude that the occurrence of TACS3 alone in our model cannot fully account for observed survival rates among patients with breast cancer [18]. Factors conducive to tumour fitness and adaptability concurrent with TACS3, such as EMT, are necessary to explain observed survival trends, and our model suggests that cancer phenotypic adaptive mechanisms significantly contribute to diminished survival outcomes (electronic supplementary material, figure S10B–C). This further substantiates our earlier hypothesis regarding the concurrence of EMT and TACS3. Under this assumption, we also compared the tumour evolution trajectories between TACS1→TACS2 and TACS1→TACS2→TACS3 conditions with varying TCR recognition abilities; representative results are shown in electronic supplementary material, figure S12.

#### 3.3.2. Large-scale stochastic simulations predict tumour-associated collagen signature 2 to have greater reductions in overall survival with immune checkpoint up-regulation, in addition to more effective responses to checkpoint blockade therapy

Given the impact of TACS and phenotypic adaptation on altered immunogenicity and subsequent expected cancer escape, along with the ability of mesenchymal tumour cells to upregulate PD-1 (programmed cell death protein-1)/PD-L1 (programmed death ligand-1) expression by modulating certain pathways [53], we further explored how TACS-specific tumour evolution affects responsiveness to PD-1/PD-L1 inhibition and its subsequent impact on simulated survival. We next considered an increase in PD-1/PD-L1 levels at a fixed time point, which acts in our model to make T-cell recognition of tumour cells more difficult by requiring additional recognition capacity of tumour antigens to overcome the checkpoint. We then simulated the dynamics of tumour–immune interactions using the calibrated *in silico* patients that previously resolved survival probabilities in TACS2 and TACS3 (electronic supplementary material, figure S11).

In our model, immune checkpoint upregulation raises the threshold for T-cell recognition of TAA. While there are multiple ways to account for this, we decided to allow cancer cells to become eliminated and recognized only if at least two TAAs were recognized since this uniformly impairs all T-cell recognition in the presence of immune checkpoint. Building on electronic supplementary material, figure S11A, we introduced PD-L1+ into TACS2 and TACS3, respectively (electronic supplementary material, figure S13). We found that (i) the introduction of PD-L1 negatively affects the survival rate for TACS2 more than for TACS3 (electronic supplementary material, figure S13A,B). (ii) Despite this, TACS3/EMT + consistently shows the lowest survival among all combinations (electronic supplementary material, figure S13C–E). (iii) Specifically, in the TACS2/EMT−/PD-L1− and TACS3/EMT+/PD-L1 + conditions, we obtained maximally resolved survival curves in large-scale stochastic simulation (electronic supplementary material, figure S13F). These results demonstrate that PD-L1 status can further exacerbate the likelihood of tumour-immune escape.

**Figure 7.**
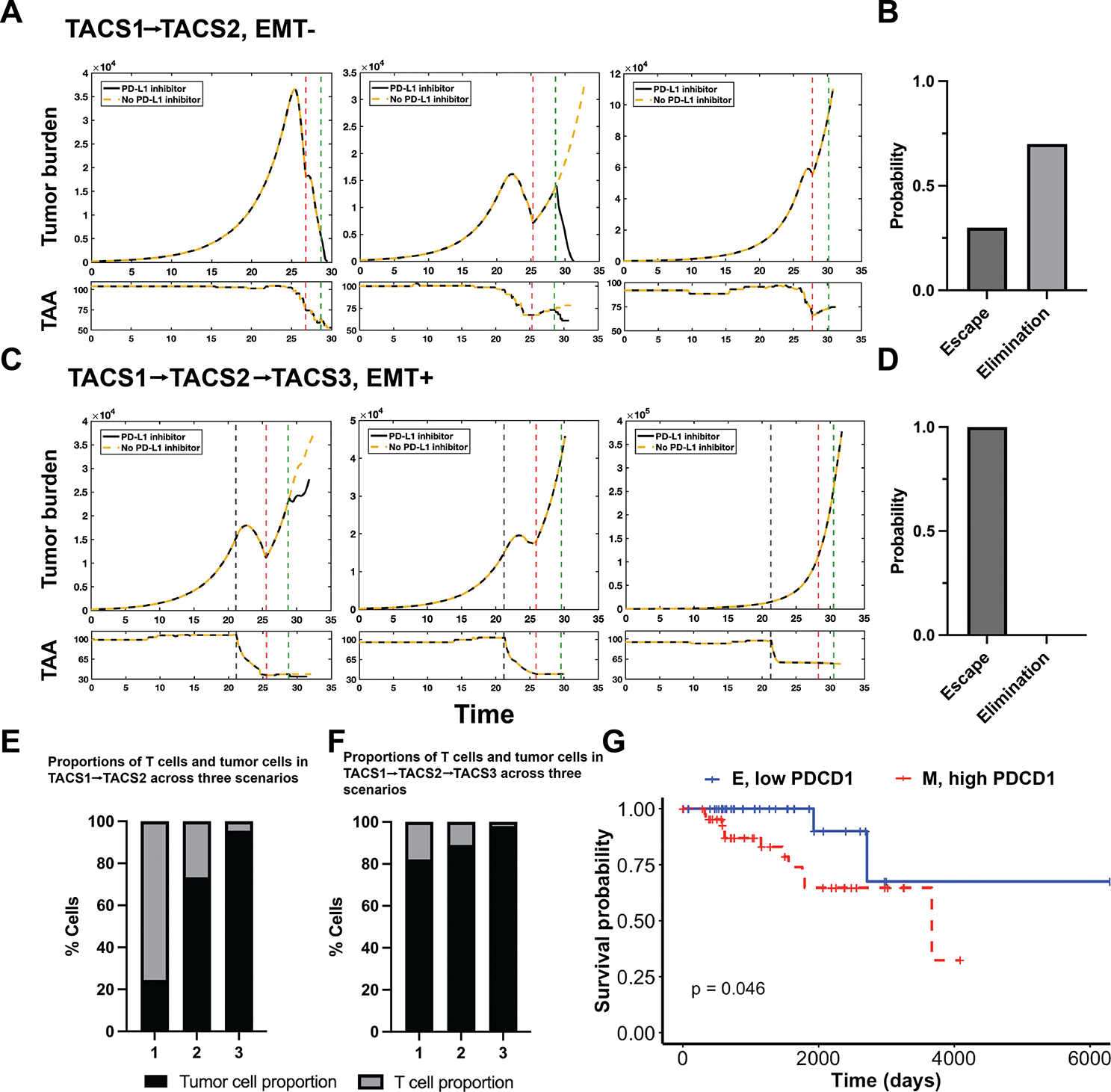
TACS-specific tumour evolution affects checkpoint inhibitor efficiency. (A) Representative tumour burden dynamics and associated mean TAA counts in the TACS1→TACS2/EMT− conditions are depicted using identical model parameters. The red and green dashed lines represent the time of PD-L1 elevation and the administration of the PD–1/PD-L1 inhibitor, respectively. Three representative images are shown from left to right, illustrating three scenarios: (1) inhibitor responder, where tumours are eliminated regardless of inhibitor administration; (2) inhibitor responder, where inhibitor administration leads to tumour elimination, while without inhibitor, tumour escape occurs; (3) inhibitor non-responder, where tumours escape both with and without inhibitor administration. (B) In the TACS1→TACS2/EMT− condition, the probability distributions of tumour elimination and escape after the addition of the inhibitor are depicted. (C) Under the same parameter regime, the representative tumour burden dynamics and associated mean TAA counts in the TACS3/EMT+ conditions are depicted. The grey dashed line represents the time of EMT occurrence. Two red dashed lines represent the time of PD-L1 elevation and the administration of the PD–1/PD-L1 inhibitor, respectively. In all three scenarios, tumours all escape. (D) In the TACS1→TACS2→TACS3/EMT+ condition, the probability distributions of tumour elimination and escape after the addition of the inhibitor are depicted. (E,F) In both aforementioned settings, the proportions of tumour and proliferating T cells at the pre-treatment time point are depicted. (G) The overall survival probability of two cohorts, E with low-PDCD1 expression and M with high-PDCD1 expression, in the TCGA BCA database were analysed. Refer to §5 for more details. In all conditions, λ=0.2, $\alpha_{t}=0.01$, $\alpha_{T}=1.8$, Q=500, q=100.

We next wanted to investigate the potential impact of TACS on checkpoint inhibitor responsiveness. We conducted 10 replications each for TACS1→TACS2 and TACS1→TACS2→TACS3 conditions. In our model, introducing the inhibitor represents the restoration of TCR recognition capability to its pre-elevated state, keeping all other parameters consistent. We then tracked the population dynamics of both tumour burden and mean TAA availability. Representative images are depicted in figure 7A,C. In our repeated experiments, TACS1→TACS2/EMT− resulted in variable dynamics, including both tumour elimination and escape (figure 7A,B). Through an examination of the antigenic level of tumour clones, we found differing levels of immunoediting. Conversely, we consistently observed tumour escape in the TACS1→TACS2→TACS3/EMT + setting, indicating either non-responsiveness or a lack of highly effective responses (figure 7C,D). Here, EMT reduces the effective number of TAAs available for T-cell targeting. The addition of PD-L1 overexpression further exacerbates this effect, presenting a considerable obstacle to the immune response. In the presence of a substantial tumour burden, even with the addition of inhibitors, our simulations predict that there is minimal, if any, enhancement in cancer cell recognition with such treatment. We further compared the proportions of proliferating T cells before the application of PD-L1 inhibition and found that a higher proportion of proliferating T cells correlates with lower tumour burdens and favourable ultimate cancer elimination rates (figure 7E,F). This observation aligns with recent findings in triple-negative breast cancer of the importance of proliferative fractions of T cells as the second-most significant predictor of immune checkpoint blockade response, following major histocompatibility complex I and II expression [54]. Taken together, our results suggest that both PD-L1 and TACS affect the likelihood of tumour-immune escape, with PD-L1 having a greater impact on TACS1→TACS2/EMT− tumours compared with TACS1→TACS2→TACS3/EMT + tumours. Additionally, the timing of PD-L1 inhibitor use is crucial, with its effectiveness being greater when used early on in tumour progression prior to TACS3 and additional phenotypic changes [55].

To validate our findings, we conducted a survival analysis on patients with breast cancer from the Cancer Genome Atlas (TCGA) database. We utilized E-cadherin (CDH1) and Vimentin (VIM) as markers to delineate epithelial (E) and mesenchymal (M) cohorts, respectively [56]. Since TCGA lacks detailed information on TACS status directly, we assumed in these analyses that TACS2/EMT− and TACS3/EMT+ occurred concomitantly as was most consistent with our prior results. We selected samples from patients who were naive to checkpoint inhibitors. E and M gene signatures were obtained using 40 genes associated with each phenotypic state, and the survival data of samples containing the highest E and M signatures were used for analysis (electronic supplementary material, figure S14A,B; see §5 for full details) [57–59]. We compared survival differences between the two cohorts and although general trends were in agreement with our model predictions, we found no statistical significance (electronic supplementary material, figure S14C). We observed similar findings when considering up-regulated versus down-regulated PD-1 expression (encoded by PDCD1) independently (electronic supplementary material, figure S14D,E). However, when patients were simultaneously stratified according to EMT and PDCD1 status, we identified a statistically significant reduction in overall survival for patients with cancer cells having M/high PDCD1 signatures, relative to those with E/low-PDCD1 signatures (figure 7G). These findings are in agreement with our model predictions (electronic supplementary material, figure S14D,E). However, when patients were simultaneously stratified according to EMT and PDCD1 status, we identified a statistically significant reduction in overall survival for patients with cancer cells having M/high PDCD1 signatures, relative to those with E/low-PDCD1 signatures (figure 7G). These findings are in agreement with our model predictions (electronic supplementary material, figure S13F) and offer a dynamical explanation for how phenotypic adaptation and immune checkpoint together can impair T-cell recognition and treatment outcomes in checkpoint inhibitor-naive patients.

## 4. Discussion

In solid tumours, ECM topology is known to be an important feature of the tumour microenvironment that affects tumour progression, metastasis and therapeutic resistance [60,61]. However, the precise way in which cancer growth and tumour–immune coevolution are affected by this topology remains unclear. To begin to address this, we developed EVO-ACT model, to explore the dynamic and spatial aspects of immune cell and tumour interaction as a function of TACS. We applied our model to gain quantitative insights into several dynamic features of this complex process, including how TACS affects both tumour-cell and T-cell migration efficiency. Our findings suggest that TACS exert a greater impact on chemokine-driven T-cell infiltration, and they do not constitute an absolute barrier to T-cell infiltration. Our findings predict that TACS-specific T-cell infiltration patterns influence immunoediting, and TACS3, together with late-stage phenotypic adaptations, such as EMT and elevated PD-L1 expression, collectively contribute to reduced predicted patient survival and decreased responsiveness to PD-L1 inhibitors.

Our work identified the impact of TACS in cell migration direction, spatial distribution and quantified the cell invasion efficiency in three common TACS, with TACS3 > TACS1 > TACS2. The highest migration efficiency observed in TACS3 offers new insights into the enhanced cell migration observed in TACS3 compared with random fibre environments [41]. Additionally, our findings suggest that TACS do not constitute an absolute barrier to cell invasion, which has been debated in recent years [13,14,20,21]; rather, it influences cell invasion efficiency significantly. Ideally, with prolonged infiltration, sufficient T-cell penetration may also be observed within TACS2 regions (figure 4A–D). Our model permits tumour cells to migrate along aligned fibres without enforcing a preference for leaving the tumour bulk. As seen in figure 3A (second row), despite omitting other details that would encourage tumour cell outward invasion, we still observe substantial variation in migration resulting purely from different TACS3 configurations. Additionally, the variation in invasion efficiency induced by TACS differs between tumour cells and T cells. Under the directed influence of chemotaxis, our model predicts that T-cell migration is more affected by TACS than tumour extravasation. These results suggest that the density of ECM exerts varying impacts on the migration of tumours and T cells situated within different TACS (electronic supplementary material, figure S4). Reductions to ECM density in the TACS2 environment showed a potentially greater improvement in cell migration efficiency for both tumour and T cells compared with those in a TACS1 or TACS3 environment. This suggests that TACS2 might be uniquely restrictive to cell movement, making it a critical target for treatment modulation. Additionally, due to the influence of chemokine gradient, therapies targeting stromal architecture could have a more pronounced effect on enhancing T-cell infiltration than on tumour-cell migration. Our analysis indicates that T cells, which are highly responsive to chemokine cues, benefit more from reductions in ECM density, leveraging these gradients for improved navigation and infiltration within the tumour stroma. Prior work has underscored the growing recognition of the importance of ECM geometry in influencing cell invasion and deepening our understanding of the elevated invasion efficiency in aligned fibres [28,41,62,63]. Subsequent studies can build on this foundational understanding by evaluating the therapeutic potential of selective targeting of ECM remodelling in specific tumour regions and to improve immune activity within the tumour microenvironment.

Since TACS do not completely hinder T-cell infiltration, our analysis focused on highlighting the heterogeneous T-cell spatial distributions in each TACS. Prior work has illustrated the importance of including chemokine attraction and antigen specificity for generating observed T-cell spatial distributions [39]. Our model’s population dynamics demonstrate that TACS also play a significant additional role in generating observed T-cell spatial distributions. Given the significant role of TACS in allocating spatial positions for tumour clones, we argue that the spatial distribution of T-cell clones is also influenced by TACS, which in turn influences the location of expanding T-cell clones (electronic supplementary material, figure S9). An alternative theoretical approach focused on T-cell distributions in the tumour core, invasive margin and tumour stroma. Significant differences in T-cell distribution and function have been observed in these three distinct regions, with notable characteristics identified in each. T cells within the tumour core exhibit tight interactions with tumour cells due to their close proximity [64]. The prolonged stimulation from tumour antigens may be a contributing factor to T-cell exhaustion, supported by recent research demonstrating that chronic tumour antigen/TCR stimulation reinforces epigenetic programmes associated with dysfunctional hallmarks, resulting in dysfunctional T cells [39,65]. These results are consistent with prior findings that T-cell dysfunction and exhaustion correspond to higher expression of co-stimulatory molecules, such as PD-1, TIM-3 and VISTA [66,67]. This phenomenon is comparable to our model (electronic supplementary material, figure S9) in settings where T cells that have interacted with the tumour are assumed to be exhausted, leading to the concentration of potentially exhausted T cells inside the tumour. In contrast, T cells in the invasive border are believed to have higher density, enhanced functionality and weak expression of co-inhibitor molecules compared with those in the tumour core or stroma [66,68,69]. In our model, T cells migrate toward the tumour, at which point factors such as antigen specificity, inflammatory cytokines and distinct TACS lead to varying distributions of expanding T cells within the tumour. However, we found that the majority of T-cell clones are still predominantly located at the tumour margin.

Our results demonstrate that TACS modulate T-cell infiltration efficiency, which in turn influences tumour evolution through distinct levels of immunoediting. TACS-specific changes in T-cell infiltration, therefore, result in varying degrees of selective pressure on tumours. Under conditions of heightened T-cell infiltration and recognition, potent negative selective pressure drives surviving cancer cells to have reduced immunogenicity [2]. This heterogeneity of T-cell infiltration and neoantigen-derived immunoediting has been observed in various cancers, such as lung cancer and breast cancer [2–4,21], which can be achieved through loss of heterozygosity in human leukocyte antigens, depletion of expressed neoantigens, or decreased TAA presentation efficiency [2,4]. Furthermore, studies indicate that the distribution of tumour mutation burden is heterogeneous, and tumours with heterogeneous infiltration are more prone to manifesting a heterogeneous tumour mutation burden [2,34,70]. Our results corroborate these findings (electronic supplementary material, figure S15), and our *in silico* approach can be used in further studies assessing the role of TACS in inducing tumour mutation burden heterogeneity.

While we have shown that TACS may influence tumour evolution in a variety of ways, our results indicate that one potential reason for the lower survival rates observed in TACS1→TACS2→TACS3 cancer patients is the concomitant occurrence of phenotypic adaptation [18]. In particular, clinically observed survival trends in TACS1→TACS2→TACS3 and TACS1→TACS2 cases in breast cancer were only explained in our modelling framework by incorporating additional phenotypic adaptation mechanisms, including EMT and increased PD-L1 expression [18]. Our results suggest that TACS3 in isolation may not be the primary driver of lower survival rates. The co-occurrence of TACS3 with EMT provides one possible explanation wherein heterogeneous and invasive clones are facilitated by a TACS1→TACS2→TACS3 environment to simultaneously avoid immune recognition and metastasize out of the primary tumour site [12,37,41,62,71,72]. Other relevant phenotypic adaptation mechanisms may also actively affect these results, and such distinct mechanisms may occur and be of direct importance for distinct cancer subtypes. Relevant mechanisms for metastatic disease are marked by higher growth rates and lower antigenicity compared with their ancestral clones, making them more resistant to elimination [73]. Moreover, such cellular phenotypic alterations often manifest in the late stages. Otherwise, if occurring early, such as during the TACS2 phase, our model results suggest that the rapid escalation of tumour heterogeneity and the hindrance posed by TACS2 on T-cell infiltration would expedite tumour evasion rapidly (figure 6F). Our finding that PD-L1 has a variable impact on tumours in the TACS2 and TACS3 also supports this. Our results further indicate that this TACS-specific tumour evolution trajectory also influences the efficacy of checkpoint inhibitors. The different degrees of immunoediting caused by TACS affect the responsiveness of PD-L1 inhibitors [54]. Our findings also indirectly suggest that the earlier use of inhibitors may lead to better outcomes, underscoring the importance of early differentiation between inhibitor responders and non-responders.

Based on all the above explorations of the mechanistic influence of TACS on tumours and T cells, we also acknowledge that in certain cancer types, such as breast cancer, different TACS may coexist [18]. This coexistence may lead to the simultaneous presence of the effects discussed above or introduce additional spatial heterogeneity in both tumour and T cells due to the spatial heterogeneity of TACS. Our model is capable of capturing these behaviours, making it more suitable for describing tumour-immune dynamics at the individual patient level. Our foundational model makes a number of assumptions. Obviously, a number of cell types feature in the tumour microenvironment and affect T-cell recognition, including cancer-associated fibroblasts, myeloid-derived suppressor cells and regulatory T cells to name a few. This work develops a foundational model capable of linking T-cell recognition and antigen-directed immune evasion to spatial ECM geometries, an important and necessary first step for creating a more comprehensive framework capable of relating additional features. In our model, we assume that all cells at the tumour boundary undergo EMT simultaneously upon reaching the EMT threshold. Also, we assume that the adaptation rates of all tumour clones are identical. However, in reality, different tumour clones may undergo EMT and mutation or adaptation at different times and with different rates, potentially leading to larger variations in growth, migration and collective migration across distinct intratumoural subpopulations, as well as differential TACS3 remodelling. Our model does not account for the presence of exhausted T cells; T cells are either in an activated state or have died and are subsequently removed from the system. We expect that in reality exhausted T cells that persist within the tumour core provide additional hindrance to T-cell killing. It is also likely that the pro- versus anti-tumour characteristics of the immune microenvironment, which we did not model in detail here, further determine the extent to which this exhaustion occurs. The presence of exhausted T cells and their occupation of space, along with increased metabolic demands such as oxygen consumption, further exacerbate the difficulty of T-cell killing in real-life situations compared with our model. We did not incorporate oxygen or nutrient gradients into the migration dynamics in this study. However, we acknowledge that hypoxia and other chemotactic signalling cues are important factors influencing tumour-cell behaviour, spatial competition for resources and its interplay with immune infiltration. Such effects as they relate to modifying TACS-specific distribution of cell spatial arrangements is a relevant next step and the subject of follow-up work. Third, our framework models tumour growth and tumour–immune interactions in a two-dimensional space, whereas real tumour populations develop in three dimensions. Our analysis considered idealized TACS2 and TACS3 collagen arrangements for simulations when in reality a number of variable and overlapping topologies likely exist. The incorporation of patient-specific ECM orientation is an important next step and the focus of future research efforts. Finally, the current model only considers the interaction between tumour and T cells, yet many other features in the tumour microenvironment affect T-cell recognition, including dendritic cells, tumour-associated macrophages and metabolic and chemical signatures. Nonetheless, our foundational model is able to provide a population dynamical explanation for a variety of relevant characteristics of the tumour–immune interaction.

Taken together, our results quantify the impact of ECM architecture on the tumour–immune interaction, and our modelling approach represents a novel mathematical framework to incorporate collagen-specific information into a description of tumour and immune coevolution. We also speculate that TACS may affect all elements in the tumour microenvironment reliant on fibre movement or requiring ECM penetration, potentially influencing tumour evolution or treatment outcomes. Hence, our findings also indirectly affirm the importance and necessity of adopting stroma-modifying treatments in clinical practice. Moreover, since ECM architecture does not exist independently, further research is required to investigate the collective effects of various TACS byproducts, such as angiogenesis, spatial distribution, interactions of different cells and varying microenvironments, on the tumour–immune interaction.

## 5. Methods

### 5.1. Initial conditions and model structure

All the simulations are performed using the EVO-ACT framework developed in this study. The implemented program utilizes Gillespie’s simulation algorithm to model stochastic events for two agents: tumour and T cells. Tumour-cell events include invasion, division and migration, each governed by rates $\gamma ,\;\lambda ,\;\alpha_{t}$, respectively. Successfully divided cells undergo adaptation based on the adaptation rate μ per cell division. Initially, tumour cells are assumed homogeneous. T cells only perform movement, with a rate $\alpha_{T}$. To improve computational efficiency and focus our analysis on the invasive behaviour of tumour cells, we defined a central circular region (with a radius of r) within the region of interest (with a radius of R), where tumour cells (n~4000) are initialized but not individually tracked. Only cells that migrate beyond this central core are explicitly modelled as agents. This design allows us to concentrate on the dynamics of tumour invasion, immune evasion and phenotypic evolution in the tumour–stroma interface, while maintaining reasonable computational complexity. We refer readers to electronic supplementary material, figure S16, for simulations comparing cases with and without a predefined central tumour region. Initially, we assume that 200 tumour cells have invaded the central mass. T cells ($N_{T}=5000$) are initially positioned at the region of interest boundary and commence infiltration towards the tumour, as illustrated in electronic supplementary material, figure S2. Additionally, in the current version of our model, time is represented in a non-dimensional form. This approach allows us to explore the relative dynamics between key biological processes without assuming a fixed absolute timescale. As the Gillespie algorithm inherently simulates stochastic time intervals between events, the actual time step size is dynamically determined based on the total event propensities at each simulation step. Some important parameters are listed in electronic supplementary material, table S1.

### 5.2. Tumour-associated collagen signature generation and remodelling

Collagen fibres occupy the annular space between the central tumour and the surrounding region of interest. Fibre density is imposed along a linear gradient maximal at the tumour boundary [12]. The lengths of these fibres follow a normal distribution (mean μ=10μm; variance $\sigma^{2}=\mu /10$). We remark that fibre length may vary across different tumour types and regions within the tumour. In this study, we used a previously reported general fibre length observed in tumour environments as the mean value, and generated a distribution of fibre lengths [29]. Initially, all fibres are randomly packed, and their directions are normally distributed, forming TACS1. Remodelling is accounted for by allowing cancer cells at the tumour boundary to change the fibre orientation. With each tumour-cell division, fibres within a radius of 75 μm are remodelled into perfectly aligned TACS2. The remodelled fibres will orient perpendicular to the line linking the dividing cell’s centre and the centre of the fibre undergoing remodelling. During EMT concurrent with TACS3, cells on the tumour periphery remodel fibres within a radius of approximately 0.18 cm into perfectly aligned TACS3. This remodelling is based on previous findings suggesting that tumours can alter fibres within a range of five times the original tumour sphere diameter into TACS3 [33]. We also assume that once fibres are remodelled into TACS3, they cannot revert to TACS2. In our model, TACS1, TACS2 and TACS3 represent predefined static ECM environments with decreasing levels of fibre alignment from the tumour core outward. To simulate ECM evolution during tumour progression [12], we also constructed dynamic environments composed of sequential transitions: TACS1→TACS2 and TACS1→TACS2→TACS3. These correspond to clinically observed TACS3-negative and TACS3-positive conditions, respectively [18].

### 5.3. Tumour division

We consider population dynamics for individual clones in the population. Specifically, when tumour division occurs, we first calculate the total rate of division for each tumour clone based on its size and division rate. Subsequently, we uniformly select a cell at random for division from any tumour clone according to its total division rate, ensuring that the chosen position for division does not overlap with any existing tumour cell. During each division time window, we allow a maximum of 100 cells to attempt division, with each cell having up to 200 attempts to select a division position. Upon successful division, the newly divided cell remodels fibres within a range of 75 μm from TACS1 to perfectly TACS2. Following division, each newly divided cell undergoes adaptation based on the adaptation rate μ.

### 5.4. Tumour adaptation

We assume that each tumour clone possesses a certain quantity of TAAs. For foundational understanding, this follows a Poisson distribution with a mean of 100. Each tumour cell could diversify into different clones expressing distinct TAAs at the adaptation rate μ [7,8]. Adapted cells randomly adjust the quantity of antigens, either increasing or decreasing. The variance in adjusted antigen levels also follows a Poisson distribution with a mean of 10. The division rate of the adapted tumour clone will increase or decrease by 1% of λ with each gain or loss of a TAA. Conversely, if no adaptation occurs, the new tumour cell retains the same properties as the original tumour cell, including division rate, migration rate and antigen set.

### 5.5. Tumour migration

When tumour migration occurs, we compute the migration probability for each tumour clone, akin to calculating the division probability. Subsequently, we randomly select a tumour clone based on its migration probability and then randomly choose a cell from within that clone for migration. Each tumour cell randomly selects a fibre within a range of 75 μm as the migration direction. There is an equal probability of choosing either the selected fibre’s direction or its opposite direction. The distance travelled by the tumour cell is determined by the diffusion coefficient D and the current time window (Δt). Each step in the EVO-ACT model checks for issues related to spatial overlap to ensure that neither the migration path nor the destination of each tumour cell overlaps with any other tumour cell.

### 5.6. Epithelial-to-mesenchymal transition

In our model, the initiation of EMT depends on the tumour burden. Once the cumulative tumour burden exceeds a specified threshold, which varies in each condition, all dividable tumour cells undergo EMT simultaneously. EMT induces changes, including a fivefold decrease in tumour division rate λ, a fivefold increase in migration rate $\alpha_{t}$ and a reduction in tumour clone immunogenicity, resulting in a random decrease in tumour antigen quantity following a Poisson distribution with a mean of 15. Moreover, all cells undergoing EMT will remodel fibres within a range of approximately 0.18 cm to TACS3.

### 5.7. T-cell migration, killing and expansion

To ensure sufficient immune pressure and enable meaningful analysis of tumour–immune spatial dynamics, the system was initialized with a higher number of T cells than cancer cells. We assumed that all T cells had already arrived at the tumour site at the start of the simulation, with no additional recruitment over time. When T-cell migration occurs, we randomly select a T cell for migration. The direction of T-cell migration is influenced by both the gradient of tumour-secreted chemokines and the orientation of surrounding fibres within a range of 80 μm. The migration distance of the T cell depends on the intensity of the chemokine gradient, the local fibre density and the duration of the current time window (Δt). We ensure that neither the migration path nor the destination of the T cell overlaps with any other T cell. To model the specificity of individual TCRs, we assume that each T cell can recognize only one type of antigen. If migration is successful, the T cell checks all tumour cells within a range of $\epsilon_{k}=45\;\mu m$ for a recognizable antigen. The number of checking attempts is proportional to the duration of the current time window (Δt). If a recognizable antigen is found within $\epsilon_{k}$, the T cell initiates killing. The killed tumour cell is removed from the system, and the T cell executing the killing generates a new T cell to occupy the position of the dead tumour cell. The newly generated T cell inherits the same TCR as its parent T cell. However, unlike its parent cell, all newly generated T cells have a survival window, whereas those T cells present in the model from the beginning do not possess such a survival window. Although memory cells are not explicitly incorporated into the model, the above dynamics create an effective memory of antigen-specific T cells while also accounting for the dynamics of T-cell expansions and contractions. We assume that all T cells with survival windows have equal survival periods, and their timers start upon birth. Once their designated survival period elapses, they are assumed to have died and are removed from the system, as depicted in electronic supplementary material, figure S1.

### 5.8. Clonal diversity index

To measure clonal diversity, we used the inverse Simpson index in equation (5.1):

(5.1) $$D=1/\sum_{i}{\left(p_{i}\right)}^{2},$$

where $p_{i}$ is the frequency of the ith combination of driver mutations.

### 5.9. Kaplan–Meier survival analysis and curve fitting

We defined clinical immune escape as occurring when the tumour reaches a diameter of approximately 0.5 cm (corresponding to approx. 110 000 tumour cells) and recorded the progression time. Given the diversity of individual T-cell repertoire, 50 *in silico* patients were divided into five groups, each comprising 10 individuals. Each group shared the same TCR specificity, represented by Q and q, with all other parameters constant. Identical specificities were compared between TACS2 and TACS3 groups. Initially, we conducted multiple trials with q ranging from 50 to 500 and Q from 500 to 2000, consistently finding that the survival rate of TACS3 exceeded that of TACS2. Then, we introduced phenotypic changes-EMT. To find the most representative fit to the empirical survival curves (electronic supplementary material, figure S11B), we further divided the whole population into n subgroups, with distinct specificities given for each group. This process was repeated for increasing n, until the relative fit between the experimentally observed and predicted survival curves did not improve with further subdivision into more groups. See electronic supplementary material, table S2, for specific group compositions. We utilized $L^{1}$ distances for quantifying differences in predicted $f_{p}$ and clinical $f_{c}$ curves on time interval $\left[t_{0},t_{1}\right]$:

(5.2) $$d\left(f_{p},f_{c}\right)={\left\|f_{p}-f_{c}\right\|}_{1}=\int_{t_{0}}^{t_{1}}\left|f_{p}-f_{c}\right|dx.$$

Best fits were selected based on minimizing equation (5.2).

### 5.10. Identification of epithelial-to-mesenchymal transition-associated gene signature

In the TCGA breast cancer database, based on drug treatment data, we selected 610 patients who were naive to PD-L1 inhibitors and identified the 40 genes most correlated with E-cadherin (*CDH1*) and Vimentin (*VIM*) using Spearman correlation analysis [56]. These genes were chosen as the gene signatures for the E and M groups. Building upon previous research indicating the presence of hybrid phenotypes within E and M categories [69], we defined the E and M cohorts based on the expression levels of the gene signatures in the selected 610 cases (electronic supplementary material, figure S14A,B). The cutoff values were determined by equations (5.3) and (5.4). We selected samples with simultaneous high expression of E gene signature and low expression of M gene signature as the E cohort. Conversely, samples with simultaneous low expression of E gene signature and high expression of M gene signature were chosen as the M cohort. This was performed to minimize the risk of selecting E and M signatures that contained hybrid E/M intermediate phenotypes [57,58,69].

(5.3) $$C_{E}=\overset{‾}{E}+\sigma_{E},$$

(5.4) $$C_{M}=\overset{‾}{M}+\sigma_{M},$$

where $C_{E}$ and $C_{M}$ are the cutoff values of E and M cohorts, respectively, $\overset{‾}{E}$ and $\overset{‾}{M}$ are the mean expressions of E and M gene signatures respectively, $\sigma_{E}$ and $\sigma_{E}$ are the standard deviations of E and M gene signature expressions, respectively.

### 5.11. Identification of PDCD1 high and low expression cohorts

After distinguishing the E and M cohorts, we calculate the median of *PDCD1* gene expression in these two cohorts as the cutoff value (electronic supplementary material, figure S14D). Subsequently, we further divide the E and M cohorts into *high-PDCD1* and *low-PDCD1* groups based on *PDCD1* expression.

## Supplementary Material

SI

Electronic supplementary material is available online at https://doi.org/10.6084/m9.figshare.c.7828947.

## Data Availability

The EVO-ACT model and raw data are publicly accessible via Zenodo [74]. Supplementary material is available online [75].
