# Supplementary material for: Distinct evolutionary patterns of tumour-immune escape and elimination determined by extracellular matrix architectures
Source: J R Soc Interface. Author manuscript; Available in PMC 2025 Aug 9. (PMC12312568; doi:10.1098/rsif.2025.0116)
Supplement: SI [file NIHMS2098396-supplement-SI.pdf]

Supplementary Information: Distinct evolutionary patterns of tumor immune escape and elimination determined by ECM architectures

Yijia Fan and Jason T. George

Variables	Notations	Values	References
Radius of central tumor circle	r	$5 \times 10^{-2} cm$	
Radius of ROI	R	$0.4cm$	
Tumor cell diameter	d_t	$15\mu m$	(72)
T cell diameter	d_T	$8\mu m$	(73)
Tumor division rate	λ	$0.1 - 2$	
Tumor migration rate	α_t	$0.01 - 1$	
Tumor adaptation rate	μ	$5 \times 10^{-4} - 8 \times 10^{-4}$	
Number of tumor cells in the central tumor circle	n	~ 4000	
Initial number of T cells	N_T	5000	
T cell migration rate	α_T	$1.6 - 1.8$	
Mean antigen counts in tumor clones	A	100	
Total number of fibers in ROI	N_f	8000	
Mean fiber length	l	$10\mu m$	(27)
TCR clone counts	Q	variable	
TAA-specific clone counts	q	variable	
TACS2 remodeling radius	r_2	$75\mu m$	
TACS3 remodeling radius	r_3	$0.18cm$	
The radius for a tumor cell to randomly pick a nearby fiber for movement	ϵ_t	$75\mu m$	
The radius for a T cell to randomly pick a nearby fiber for movement	ϵ_T	$80\mu m$	
The radius for a T cell to randomly pick a nearby tumor to kill	ϵ_k	$45\mu m$	

Supplementary Table S1. Values of key parameters in the model and associated references.

Variables	Group	Sample counts	q	Q
Fig 5C	G1	10	100	500
	G2	10	250	1250
	G3	10	300	1500
	G4	10	450	1800
	G5	10	500	2500
TACS2/EMT- condition in Supplementary Figure S11A	G1	14	50	1000
	G2	1	250	1250
	G3	1	300	1500
	G4	2	300	2200
	G5	5	450	1800
	G6	26	450	900
	G7	1	500	1250
TACS3/EMT+ condition in Supplementary Figure S11A	G1	2	50	1000
	G2	3	250	1250
	G3	9	250	2273
	G4	2	450	1800
	G5	6	450	2250
	G6	20	500	500
	G7	7	500	625
	G8	1	500	5000

Supplementary Table S2. The composition of different TCR specificity groups in survival plots.

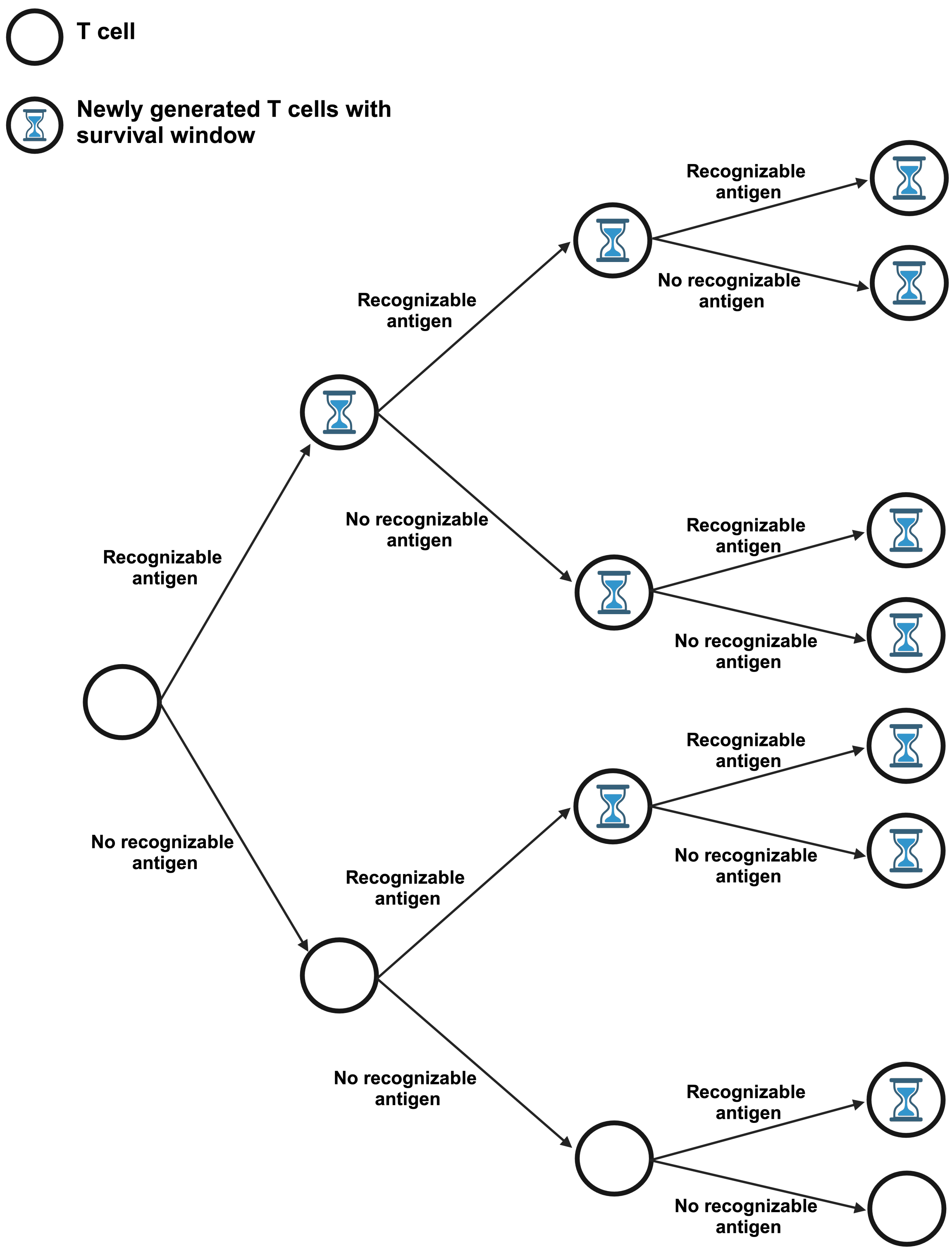

Supplementary Figure S1. Rules for T Cell expansion. In the designated region of interest, existing T cells will expand upon encountering cognate tumor antigens. These newly generated T cells share identical lifespans, starting a countdown timer upon birth. Upon its expiration, these T cells are removed from tracking.

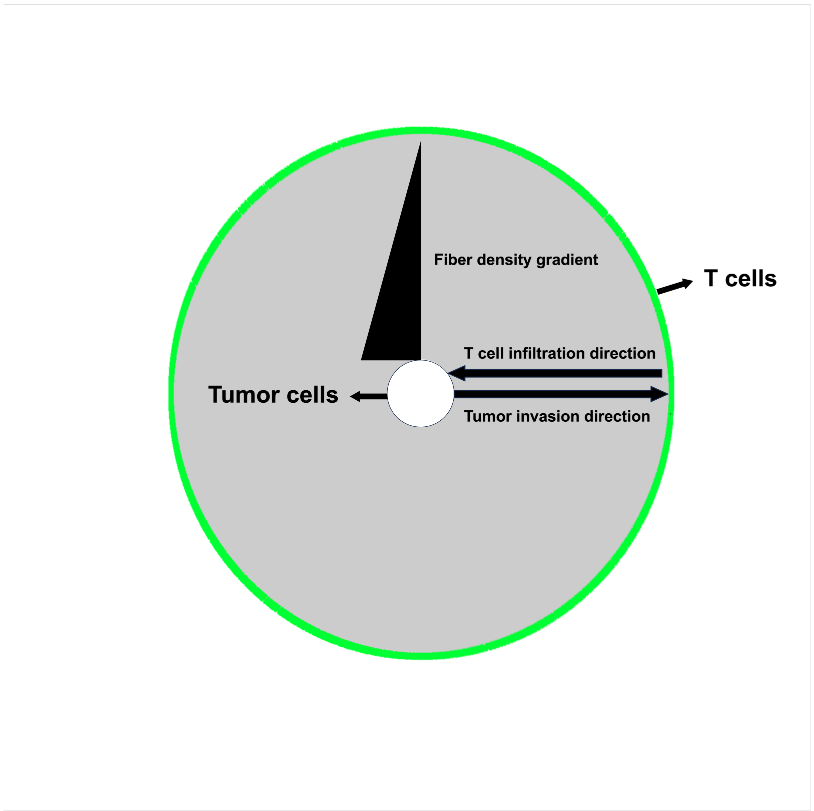

Supplementary Figure S2. The initial spatial configuration of tumor Cells, T Cells, and fibers in our model. The circular region represents our region of interest, within which a circular tumor region is initially defined. Tumor cells within this circular area must invade beyond its boundaries to migrate or divide. Those that invade will migrate outward. All T cells are assumed to be positioned at the boundary of the region of interest and will infiltrate towards the tumor. The gray area in the illustration denotes the presence of fibers. In the simulations to follow, we impose a decreasing fiber density that varies linearly as a function of the radius.

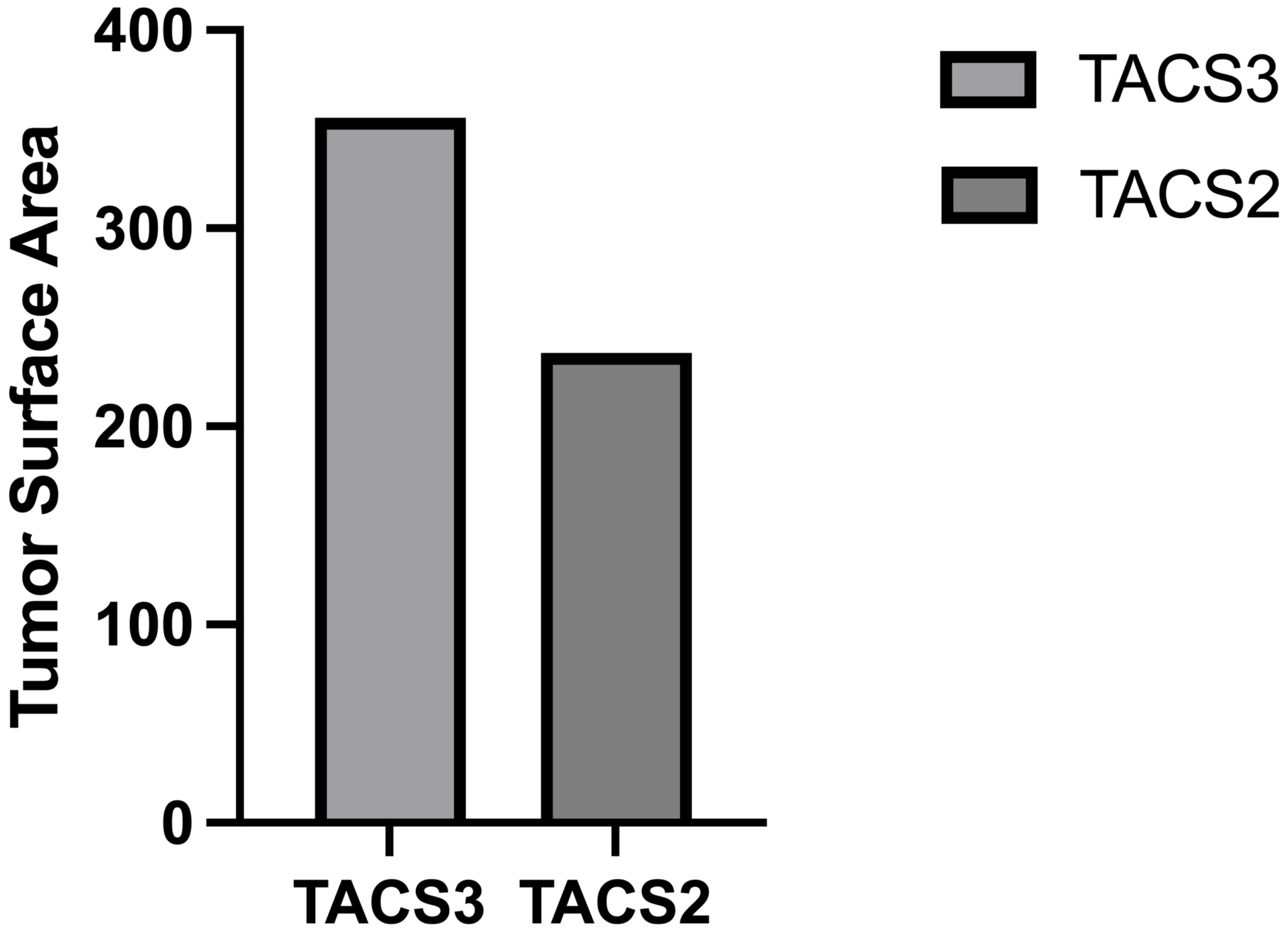

Supplementary Figure S3. Tumor boundary surface area in TACS2 and TACS3. Tumors in a TACS3 environment exhibit larger boundary surface area compared to those in TACS2, under equal tumor cell counts and simulation time from 10 independent iterations.

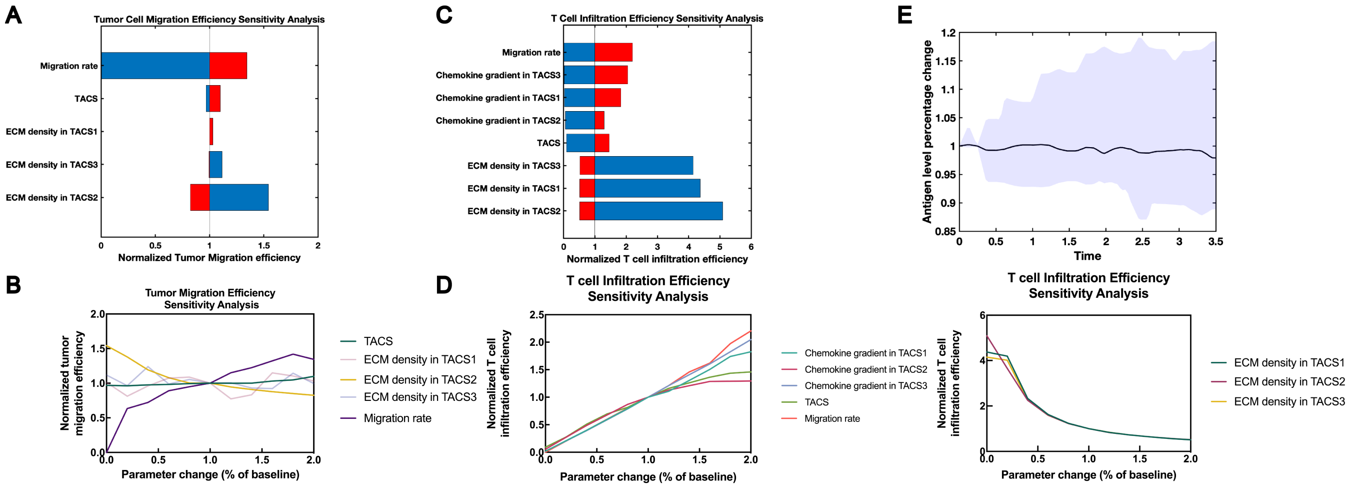

Supplementary Figure S4. Sensitivity analysis. A-B:Sensitivity analysis of tumor migration efficiency. C-D: Sensitivity analysis of T cell infiltration efficiency. E: Confidence interval plot of average antigen levels over time across different T cell infiltration levels.

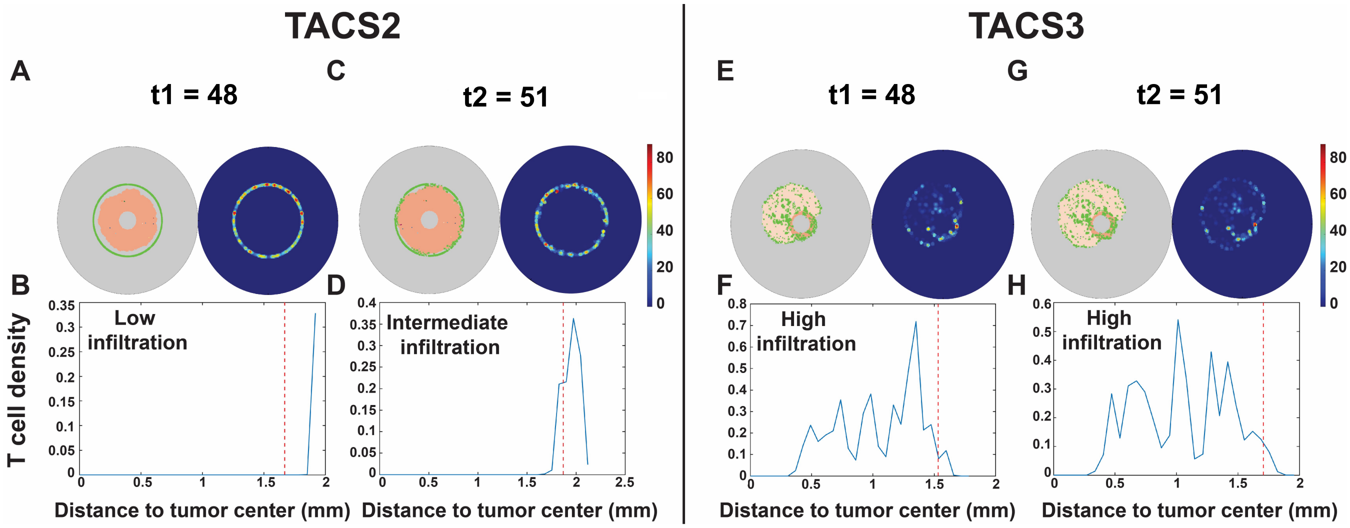

Supplementary Figure S5. TACS affects the spatial distribution heterogeneity and infiltration level of T cells. TACS3 facilitates more efficient T cell infiltration. In perfectly aligned TACS2 (A-D) and TACS3 (E-H) ($\sigma = 0$), representative spatial distributions for T cell infiltration were quantified with respect to their distance to the tumor center at the same time point t1 and t2. The red dashed lines in the graph represent the boundary of the tumor core. For the same time duration, T cells in TACS3 can infiltrate into the interior of the tumor more efficiently.

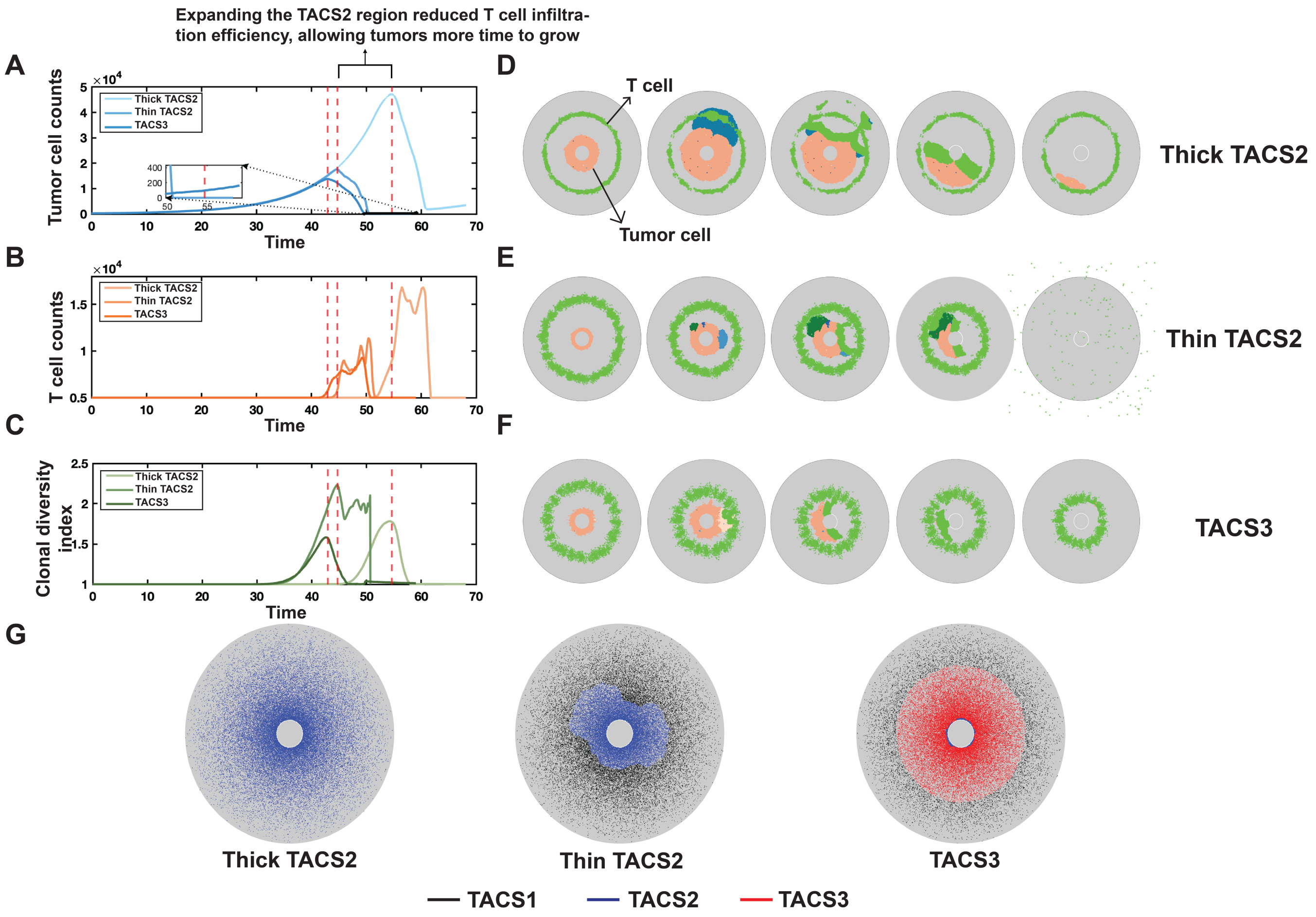

Supplementary Figure S6. Tumor and T Cell interaction dynamics in varied TACS2 and TACS3 configurations. A: Tumor burden is depicted over time. The red dashed lines represent the time at which T cells initiate large-scale tumor killing in each condition (defined by the time of maximal tumor size). B: T cell counts are depicted over time. C: Clonal diversity index is depicted over time (see Methods for full details). D-F: Snapshots of each condition at different times respectively. T cells are represented by green, while the remaining cells represent tumor cells, with different colors indicating distinct tumor clones. Among them, D: Thick TACS2 region. E: Thin TACS2 region. F: A comparison of TACS3 condition. G: Final distribution of fibers in each condition. TACS1 fibers are marked with black, TACS2 fibers are marked with blue, and TACS3 fibers are marked with red. In all simulations, $\lambda = 0.1$, $\alpha_t = 0.02$, $\alpha_T = 1.8$.

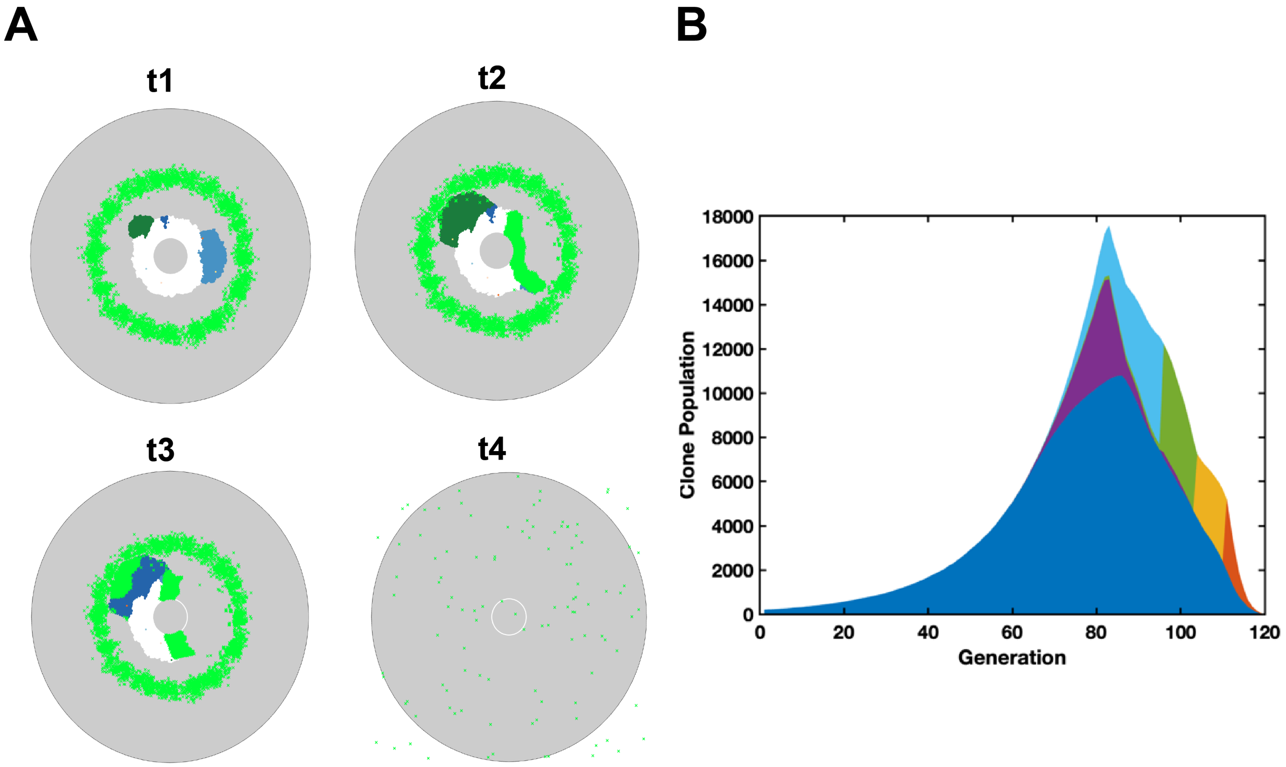

Supplementary Figure S7. Tumor progression and clonal evolution under T cell predation. A: Snapshots of tumor and T cells spatial distribution at four time points t1-t4. T cells are represented by green, while the remaining cells represent tumor cells, with different colors indicating distinct tumor clones. B: Population dynamics and proportional changes of individual tumor clones over time. Each color represents a distinct tumor clone.

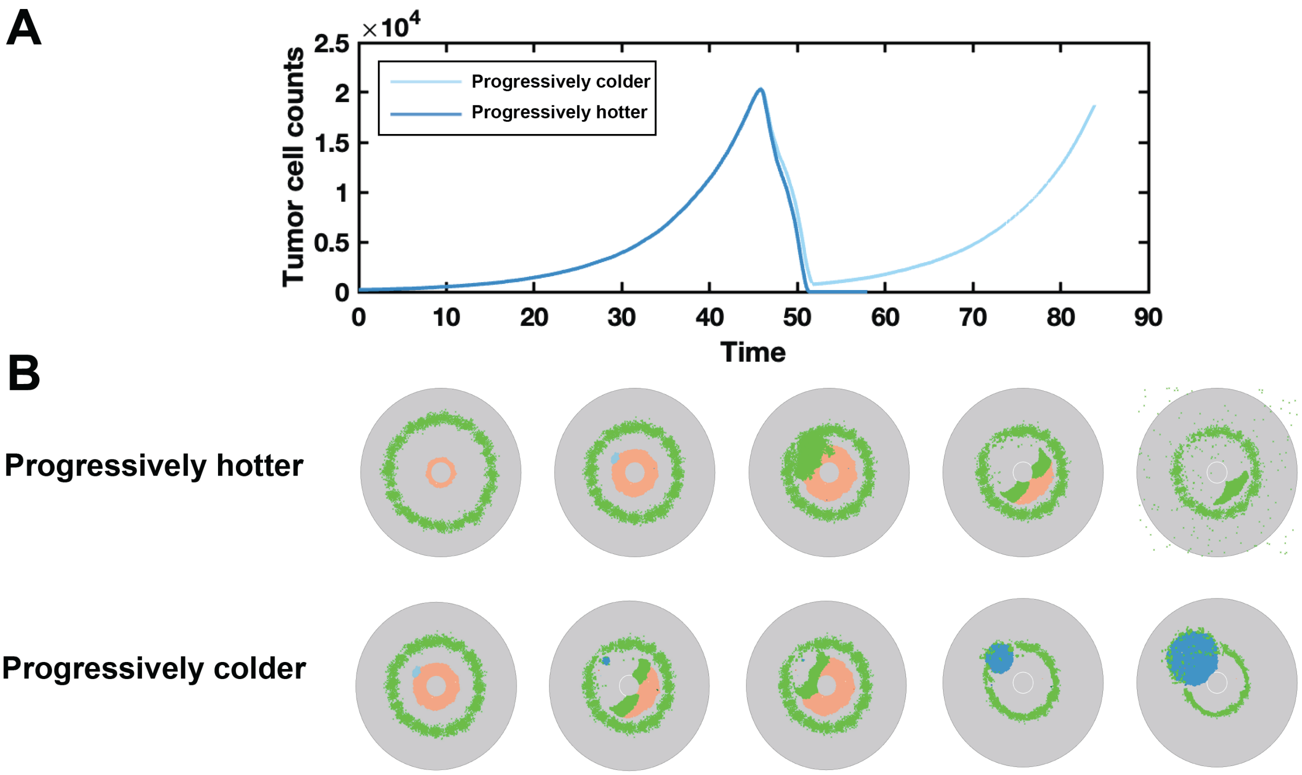

Supplementary Figure S8. The progressively colder tumor presents an escalating challenge for immune killing. We simulated two conditions and representative examples are shown above: one with increasing immunogenicity, termed "hot", and the other with decreasing immunogenicity, termed "cold". Tumors exhibiting decreasing immunogenicity pose greater difficulties for immune cell infiltration, rendering them more prone to relapse.

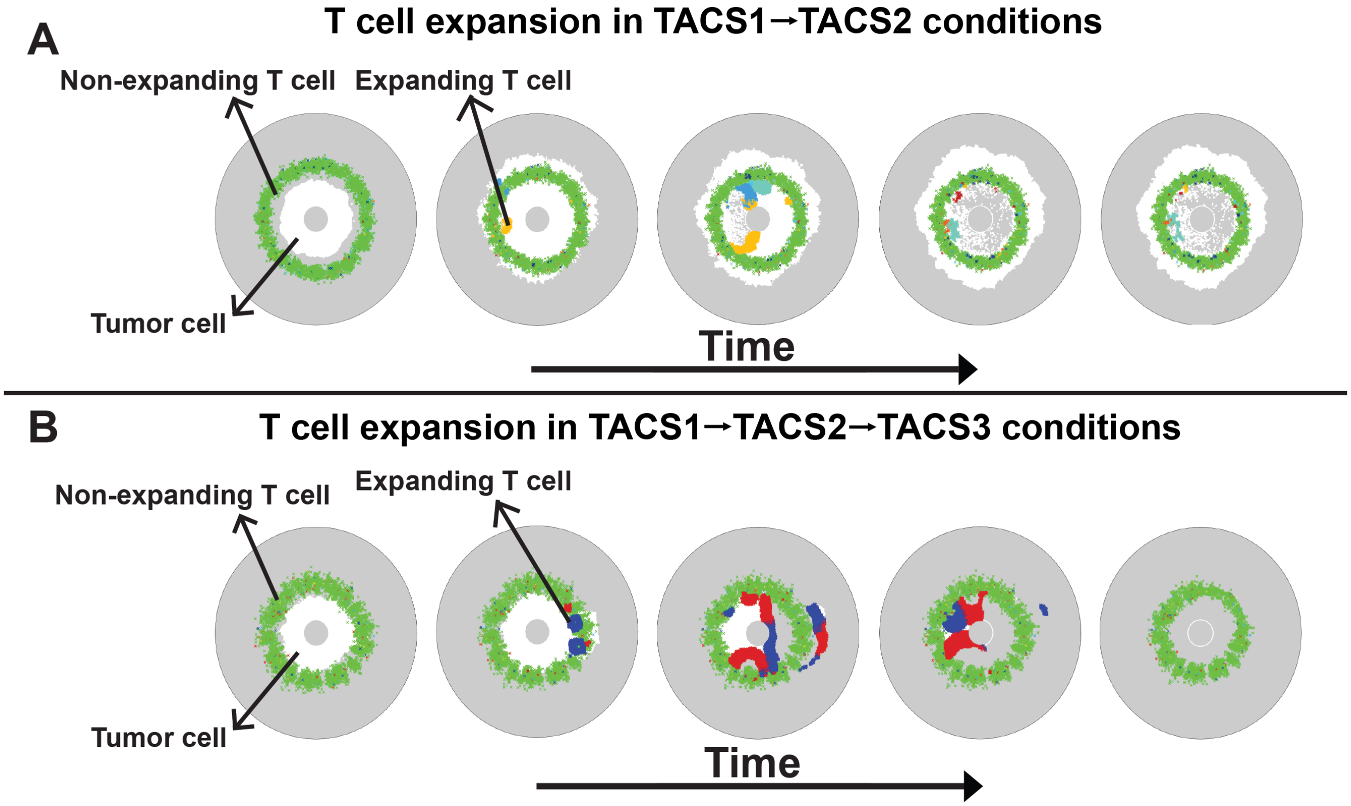

Supplementary Figure S9. TACS further influences the spatial distribution of expanding T clones by influencing the spatial distribution of tumor clones. The spatial distribution of T cell expansions against tumor clones are illustrated through time for representative cases involving A: TACS1→TACS2 and B: TACS1→TACS2→TACS3 conditions. All tumor cells are marked in white. Non-expanding T clones are represented in green while expanding T clones are marked with colors excluding green ($\lambda = 0.1$, $\alpha_t = 0.02$, $\alpha_T = 1.8$, $Q = 210$, $q = 10$ in each case).

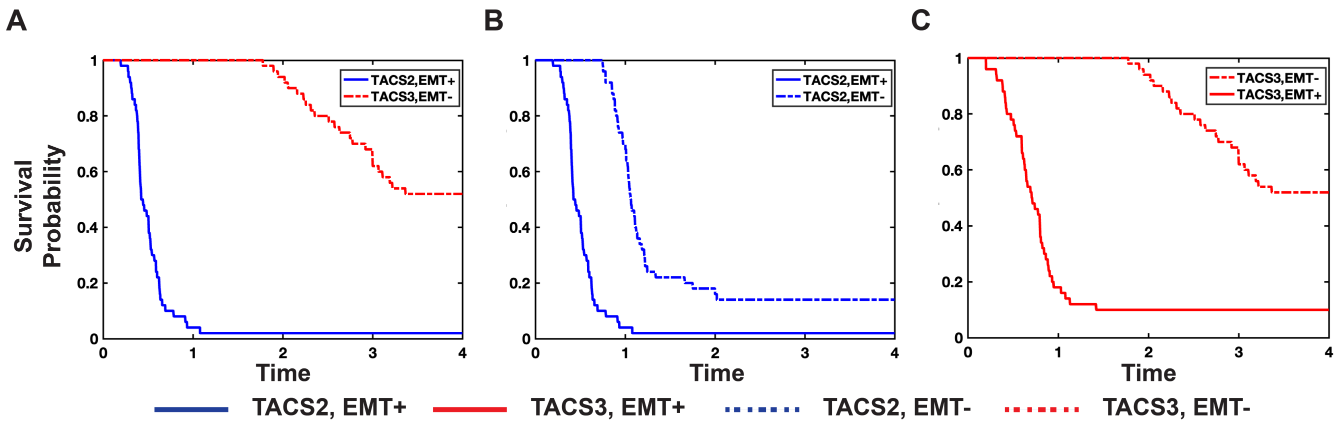

Supplementary Figure S10. TACS alone cannot account for the observed low survival rates in TACS3$^+$ cases. Elevated cell adaptation significantly reduces survival rates in both TACS2 and TACS3. The survival probability from 50 repeated experiments with varying TCR diversity in TACS2/EMT$^+$, TACS3/EMT$^-$ (A), TACS2/EMT$^+$, TACS2/EMT$^-$ (B), TACS3/EMT$^+$, TACS3/EMT$^-$ (C). In all simulations, we assumed that clinical death occurs when the tumor size reaches ~0.5cm. To simplify, we reduced the region of interest size from a radius of 0.4cm to 0.25cm. $\lambda = 0.4$, $\alpha_t = 0.01$, $\alpha_T = 1.6$, $\mu = 8 \cdot 10^{-4}$, $Q = 500 \sim 2500$, $q = 100 \sim 500$.

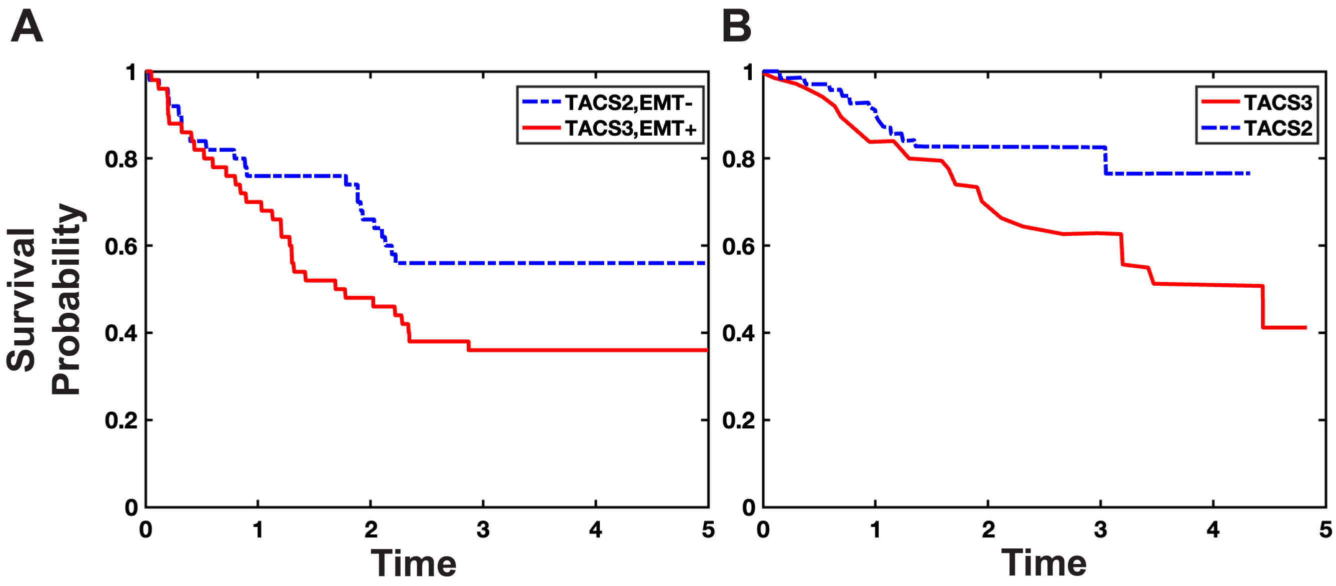

Supplementary Figure S11. Fitting of survival rate of TACS2 and TACS3 in clinically observed breast cancer survival. A: Based on Figure 5C, we further fitted the survival of clinically observed breast cancer in TACS2 and TACS3. B: Clinically observed breast cancer overall survival, the image was digitalized from (17).

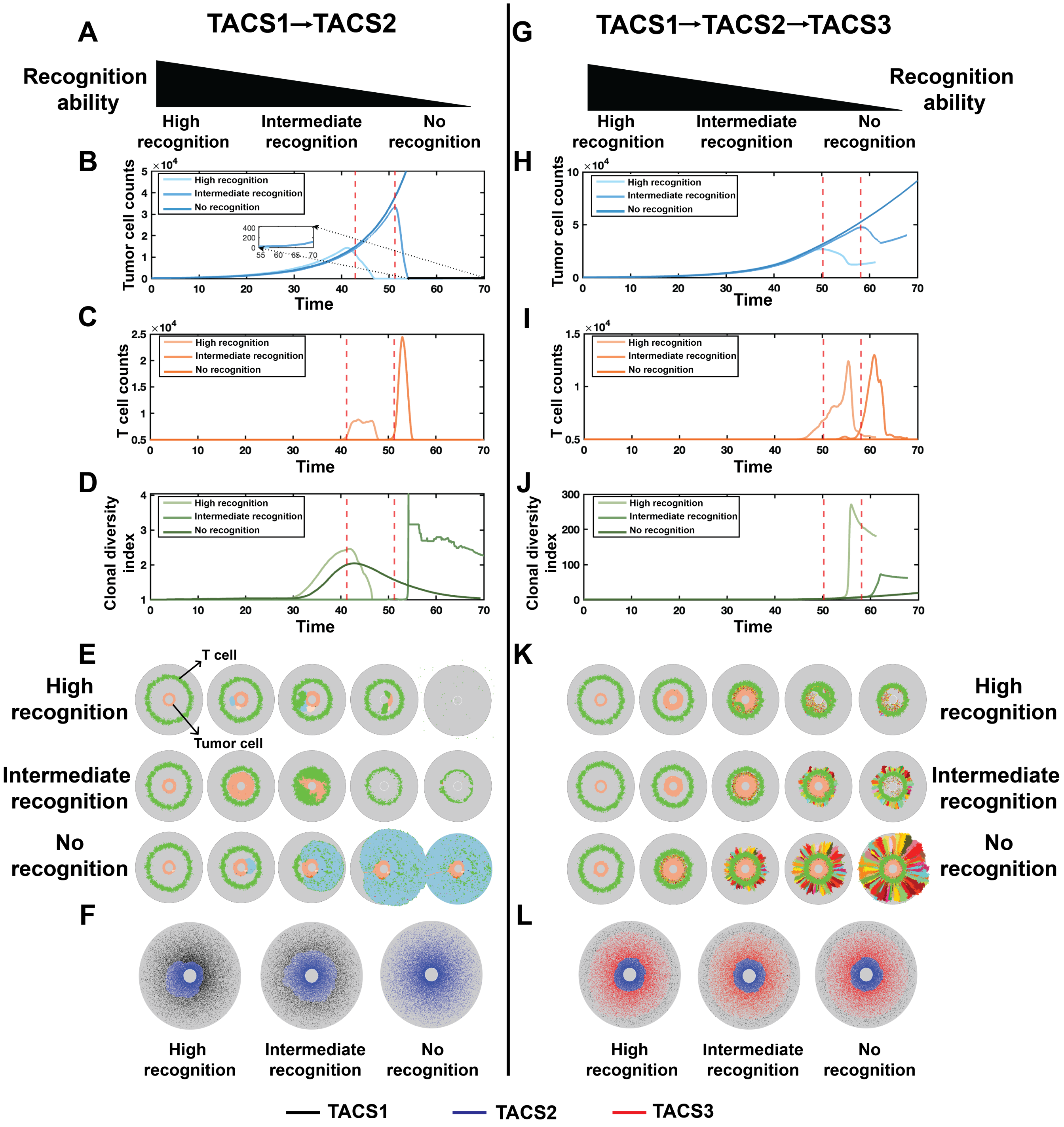

Supplementary Figure S12. Tumor-T cell dynamics during TACS transitions. A, G: In each of the three conditions for both TACS1 to TACS2 transitions and TACS1 to TACS3 transitions, it was assumed that the recognition ability of T cells gradually diminishes. B-D: Tumor burden, T cell counts, and clonal diversity index in three conditions (high recognition, intermediate recognition, and no recognition) during TACS1 to TACS2 transition are depicted respectively. Please refer to Methods for further details about the clonal diversity index. The red dashed lines represent the time at which T cells initiate large-scale tumor killing in each condition. In high recognition: $Q = 210$, $q = 210$. In intermediate recognition: $Q = 210$, $q = 10$. In no recognition: $Q = 210$, $q = 0$. H-J: Tumor burden, T cell counts, and clonal diversity index in three conditions (high, intermediate, and no) during TACS1 to TACS2 to TACS3 transitions are depicted respectively. In high recognition: T cells can recognize all tumor clones that have not undergone EMT, and some clones that have undergone EMT. In intermediate recognition: T cells can recognize all tumor clones that have not undergone EMT, and cannot recognize any tumor clones that have undergone EMT. In no recognition: T cells cannot recognize any tumor clones. E, K: Snapshots of each condition at different times respectively. T cells are represented by green, while the remaining cells represent tumor cells, with different colors indicating distinct tumor clones. F, L: Final distribution of fibers in each condition. TACS1 fibers are marked with black, TACS2 fibers are marked with blue, and TACS3 fibers are marked with red. In all conditions, $\lambda = 0.1$, $\alpha_t = 0.02$, $\alpha_T = 1.8$.

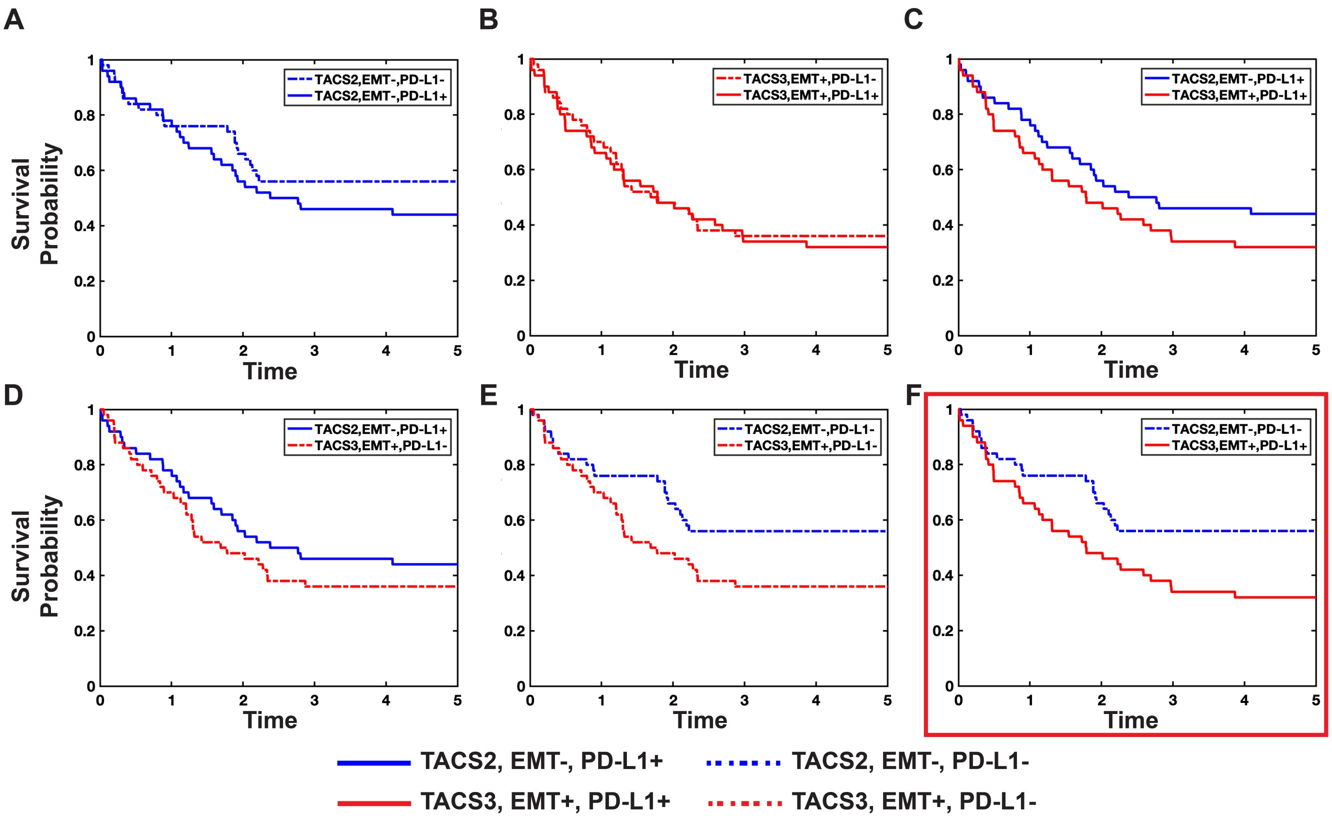

Supplementary Figure S13. Increased checkpoint expression results in decreased predicted survival rates, particularly in TACS2. Based on Figure S11, we incorporated increased PD-L1 expression in TACS2 and TACS3 respectively. The survival probability in TACS2/EMT-/PD-L1-, TACS2/EMT-/PD-L1+ (A), TACS3/EMT+/PD-L1-, TACS3/EMT+/PD-L1+ (B), TACS2/EMT-/PD-L1+, TACS3/EMT+/PD-L1+ (C), TACS2/EMT-/PD-L1+, TACS3/EMT+/PD-L1- (D), TACS2/EMT-/PD-L1-, TACS3/EMT+/PD-L1- (E) and TACS2/EMT-/PD-L1-, TACS3/EMT+/PD-L1+ (F) are depicted. In F, we found maximally resolved survival curves.

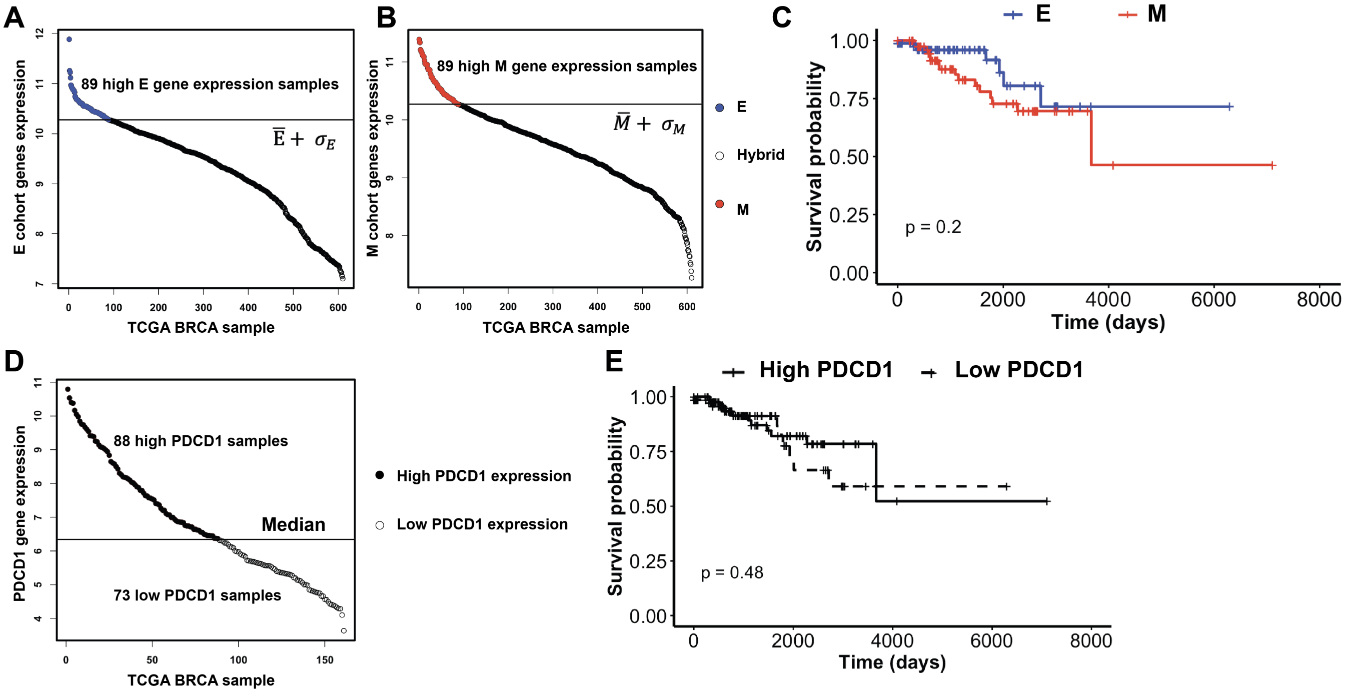

Supplementary Figure S14. Identification of E and M cohorts and PDCD1 high and low expression cohorts. A, B: In the selected 610 cases, we observed the expression status of E and M gene signatures. Based on the cutoff value, samples with expression levels above the cutoff value were selected for the E or M cohort. C: The survival probability of selected E and M cohorts, with p = 0.2. D: In the selected E and M cohorts, samples with PDCD1 expression above the median PDCD1 expression are categorized as the high PDCD1 expression group, while the remaining samples are classified as the low PDCD1 expression group. E: The survival probability of both high and low PDCD1 expression group, with p = 0.48.

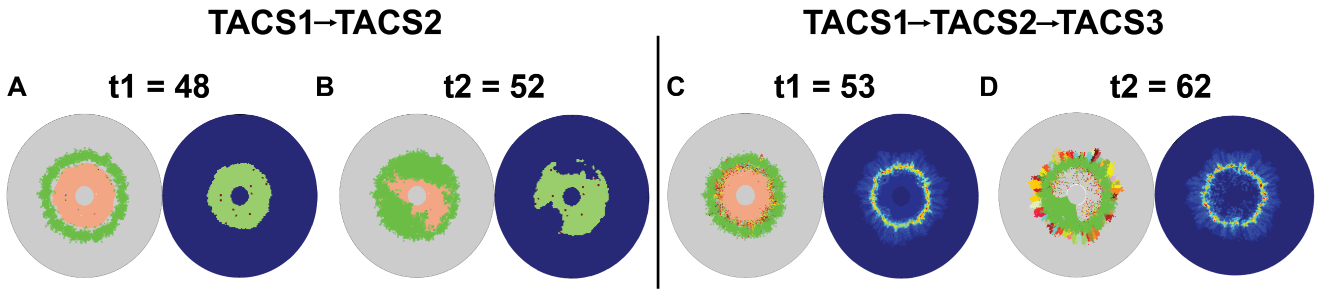

Supplementary Figure S15. The heterogeneity of TMB between TACS2 and TACS3 conditions. Under conditions identical to those in Figure 4A-H of the main text, we further quantified the spatial distribution of TMB based on the number of tumor clones surrounding each tumor at time point t1 and t2. We posit that TACS further influences the spatial distribution heterogeneity of TMB by affecting tumor spatial distribution, which is consistent with previous findings (2, 32).

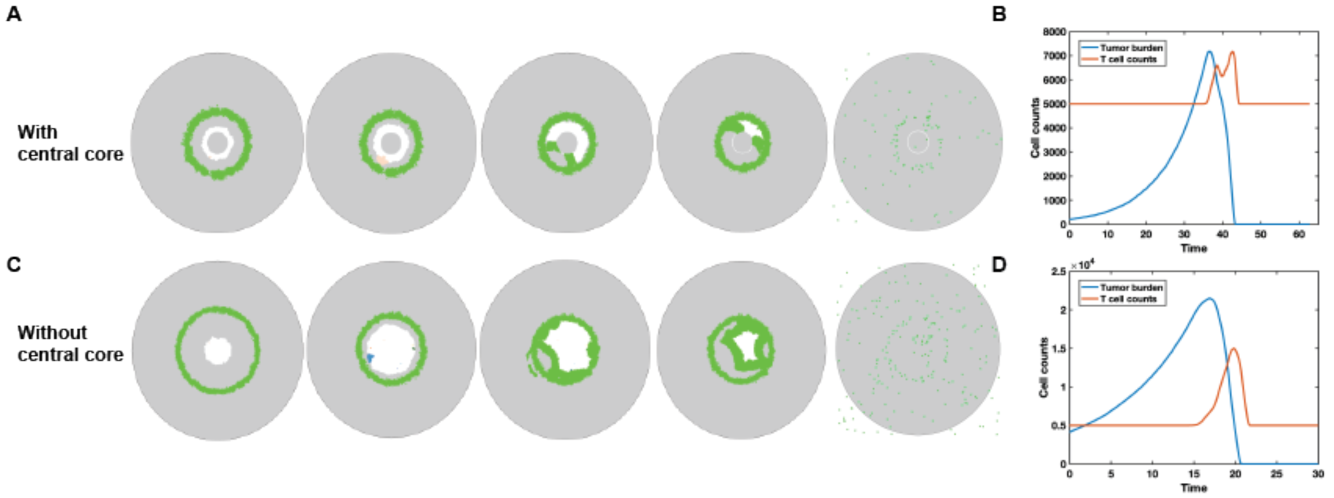

Supplementary Figure S16. Tumor progression and T cell dynamics with and without a predefined central tumor region. A, C: Representative snapshots of tumor progression at five time points under conditions with and without an assumed tumor core, respectively. B,D: Temporal dynamics of tumor growth and T cell distribution under the two conditions.
